# *Stewed Rhubarb* Decoction Ameliorates Adenine-Induced Chronic Renal Failure in Mice by Regulating Gut Microbiota Dysbiosis

**DOI:** 10.3389/fphar.2022.842720

**Published:** 2022-03-21

**Authors:** Rui Wang, Baifei Hu, Cheng Ye, Zhigang Zhang, Mingzhu Yin, Qiushi Cao, Yuanming Ba, Hongtao Liu

**Affiliations:** ^1^ College of Basic Medical Sciences, Hubei University of Chinese Medicine, Wuhan, China; ^2^ Nephrology Department, Hubei Provincial Hospital of Traditional Chinese Medicine, Wuhan, China; ^3^ Technology Center of Wuhan Customs, Wuhan, China; ^4^ Nephrology Department, Hubei Provincial Traditional Chinese Medicine Research Institute, Wuhan, China

**Keywords:** gut microbiota, intestinal metabolites, renal fibrosis, stewed rhubarb, chronic renal failure (CRF)

## Abstract

This study aimed to investigate the protective effect of *Stewed Rhubarb* (*SR*) decoction on chronic renal failure (CRF) through the regulation of gut microbiota. Using a CRF mouse model induced by a 0.2% adenine diet, we proved that *SR* decoction (2.0 g crude SR/kg) significantly reduced the levels of urea and creatinine in plasma of CRF mice, accompanied by the improvement of renal fibrosis and tubular atrophy, amelioration of inflammation, and inhibition of aquaporins damage. Also, SR decoction alleviated gut barrier damage, indicative of the elevated mRNA expression of intestinal mucins and tight junctions. By 16S rDNA sequencing, SR decoction reshaped the imbalanced gut microbiota in CRF mice by statistically reversing the abundance changes of a wide range of intestinal bacteria at family and genus levels, which further led to balance in the production of intestinal metabolites, including short-chain fatty acids (acetic acid, propionic acid, and valeric acid), indole, and bile acids (TUDCA and CDCA). Inversely, SR decoction failed to repress the occurrence of CRF in mice with gut microbiota depletion, confirming the essential role of gut microbiota in SR decoction-initiated protection against CRF. In summary, SR decoction can improve adenine-induced CRF in mice by remolding the structure of destructed gut microbiota community. Our findings shed light on the clinical application of SR decoction in nephropathy treatment.

## Introduction

Chronic renal failure (CRF) is a progressive renal parenchymal injury resulting renal atrophy and a reduction in glomerular filtration rate. The characteristics of CRF are retention of metabolites, renal fibrosis, and imbalance of fluid, electrolyte, and acid-base (Ammirati, 2020). Meanwhile, CRF is often accompanied by multiple complications, such as hypertension, nausea, and neurological disorders (Wang et al., 2016). According to statistics, the incidence rate of chronic kidney disease is 9.1%, and there are currently 697.5 million cases worldwide (GBD Chronic Kidney Disease Collaboration, 2020; Li et al., 2021). The major interventions of CRF are dialysis and kidney transplantation. However, both treatments cause a huge financial burden and usually have adverse effects, like gastrointestinal reactions, kidney transplant rejection, and dialysis complications (Wang et al., 2016).

Adenine is a purine nucleobase, which plays a vital role in the biochemical and physiological functions of cells (Dos Santos et al., 2019). Under physiological conditions, xanthine oxidase catalyzes adenine to 2, 8-dihydroxyadenine (DHA) in the liver, and DHA is finally excreted from urine (Wyngaarden and Dunn, 1957). However, the over-produced DHA will form crystals and deposit in renal tubules or interstitial tissues due to its poor solubility under the pH value of urine, leading to kidney damage (Dos Santos et al., 2019). Now, adenine is widely used to establish the experimental model of chronic renal failure (CRF) in rodents. For example, C57BL/6 mice are often fed with a 0.2% adenine diet to induce the CRF model (Mishima et al., 2015). The primary pathology of the CRF model is renal fibrosis, related to the abnormal changes of several signal pathways, such as TGF-β1/Smad, MAPK signaling, and GSK-3β/β-Catenin (Ma and Meng, 2019; Schunk et al., 2021; Zhou et al., 2021). Noticeably, GSK-3β/β-Catenin is pivotal for the formation of renal fibrosis. The phosphorylation of GSK-3β (ser9) can inhibit its enzymatic activity and suppress the degradation of β-catenin, which causes epithelial-mesenchymal transformation, renal fibrosis, and tight junction destruction characterized by the down-regulation of E-cadherin (Sun et al., 2016).

Gut microbiota interacts with various organs to maintain host health. It was shown that kidney damage is at least partly due to the dysbiosis of intestinal flora. For example, the increase of *Clostridium* and *Lactobacillales* accelerated tubular atrophy and dilatation, interstitial fibrosis, and inflammatory cell infiltration in the kidney (Chen et al., 2019). In clinical trials, the ratio of Firmicutes to Bacteroidetes and α-diversity of intestinal flora was elevated in CRF patients (Jiang et al., 2016; Chen et al., 2019). Further, the disrupted homeostasis among gut microbiota led to bacterial translocation, systemic inflammation, and subsequent renal fibrosis (Miyazaki-Anzai et al., 2021). In addition, the metabolites of gut microbiota play pivotal roles in the occurrence of CRF. In previous studies, the accumulation of uremic toxins (gut microbiota-derived metabolites) caused endothelial cell damage and microvascular injury in the kidney, followed by aggravated tubulointerstitial fibrosis (Koizumi et al., 2014; Giordano et al., 2021). In contrast, as the metabolites of intestinal flora, short-chain fatty acids (SCFAs) significantly improved renal function by reducing the production of reactive oxygen species and apoptotic cells (Wang et al., 2019). By inhibiting the biotransformation reaction of bile acids (BAs) through gut microbiota, circulating BAs were decreased, accompanied by the relief of vascular calcification and atherosclerosis in CRF (Miyazaki-Anzai et al., 2021). It seems that gut microbiota and their metabolites should be potential regulatory targets in treating CRF.

*Stewed Rhubarb* (*SR*)*,* a processed product of *Rhubarb*, has been used as a herbal medicine for thousands of years. *SR* was first recorded in “*Treatise on Febrile Diseases*”, in which *SR* was obtained by steaming raw *Rhubarb* with glutinous rice wine until it turned black (Zhu et al., 2016). *SR* has a milder purgative effect than raw *Rhubarb*, making it possible for long-term medication without significant side effects to the intestine (Yao et al., 2012). Traditional Chinese Medicine (TCM) theory believes that *SR* has pharmacological effects of defecating, relieving heat, and promoting blood circulation (Zhuang et al., 2020). These effects are attributed to multiple natural active ingredients from *SR,* including Rhein, Emodin, Aloe Emodin, Physcion, and Chrysophanol for their anti-bacterial, anti-fibrotic, and anti-inflammatory efficacy (Cao et al., 2017). So far, *SR* has been widely used to treat acute pancreatitis, constipation, and chronic renal failure (CRF) (Zhang et al., 2017; Zhang et al., 2018).

In most cases, Chinese herbal medicines are orally administered and thus will interact with gastrointestinal bacteria before exerting their pharmacological activities (Liu et al., 2020). This gives them more opportunities to affect intestinal flora and its metabolites, thus showing unique advantages in disease treatment. This study hypothesizes that SR decoction can ameliorate CRF by suppressing the imbalanced gut microbiota and their metabolite changes. Based on an adenine-induced CRF mouse model, we investigated the protective effect of SR decoction on the damage to mouse kidneys. We also examined the improvement of SR decoction on the change of intestinal flora structure and alteration of microbial metabolite profiles in CRF mice. Further, a germ-depletion mouse experiment was designed to assess the effect of SR decoction on CRF via gut microbiota modulation.

## Materials and Methods

### Reagents

*Stewed Rhubarb* (*Polygonaceae*; *Rhei Radix et Rhizoma*) (*SR*) is the dried root and rhizome of *Rheum palmatum* L. And *SR* was purchased from Hubei Tianji Chinese Medicine Decoction Company (Wuhan, China) with the batch number 202005018. Sodium butyrate, sodium acetate anhydrous, sodium propionate, valeric acid, adenine hydrochloride, Metronidazole, Ampicillin, Neomycin sulfate, Gentamycin sulfate, Gallic acid, Aloe-emodin, Chrysophanol, Physcion, Rhein, and Emodin were obtained from Aladdin (Shanghai, China). Rhein-8-O-β-D-glucopyranoside was purchased from YuanyeBio Co., Ltd. (Shanghai, China). Indole, cholic acid (CA), taurocholic acid (TCA), chenodeoxycholic acid (CDCA), taurochenodeoxycholic acid (TCDCA), deoxycholic acid (DCA), Taurodeoxycholic acid (TDCA), tauroursodeoxycholic acid (TUDCA), and Ursodeoxycholic acid (UCDCA) were obtained from Sigma (St. Louis, MO, United States). Tauro-α-murocholic acid (T-α-MCA) and tauro-β-murocholicacid (T-β-MCA) were purchased from TRC (Toronto, Canada). Primary antibodies against phosphorylation-GSK-3β (ser 9) and GSK-3β were obtained from Cell Signaling Technology Inc. (Beverly, MA, United States). Other antibodies, including β-Catenin, E-Cadherin, and β-actin, were separately purchased from Proteintech Group, Inc. (Wuhan, China), Abcam (Cambridge, MA, United States), and Santa Cruz Biotechnology (Santa Cruz, CA, United States).

### Water Extracts Preparation and Compositional Identification of SR Decoction

The preparation of SR decoction referred to the regulation of “*Treatise on Febrile Diseases*”. In brief, the drug–solvent ratio was 1:8, that is, 30 g of *SR* species (*Rheum palmatum L*; 202005018, Hubei Tianji Chinese Medicine Decoction Company, Wuhan, China) was added into 240 ml of boiling water and extracted for 10 min. Next, the SR decoction was concentrated to 150 ml, and the crude drug content of SR decoction was 200 mg/ml. After that, a part of SR decoction was dried in a vacuum drier to calculate the extraction rate, and the ratio of SR decoction powder to raw herbs was 7.31%. Based on the above, a 2 g crude SR/kg was equivalent of 146.2 mg/kg SR powder in this study. The dose conformed to the concentration range (100–200 mg/kg *in vivo* studies of extracts) (Heinrich et al., 2020). The experimental dose was obtained by a preliminary experiment (Supplementary Table S1).

To analyze the composition of SR decoction, we conducted High-Performance Liquid Chromatography (HPLC) analysis on a Waters-system (Waters Corp, Milford, United States) with an Agilent Eclipse XDB C18 column (250 × 4.6 mm, 5 μm). The flow rate was 1.0 ml/min, and the mobile phase was composed of 0.2% acetic acid v/v (A) and acetonitrile (B). The gradient elution with a flow rate of 1.0 ml/min was as follows: 5–12% B at 0–10 min, 12%–26% B at 10–28 min, 26%–38% B at 28–53 min, 38%–42% B at 53–70 min, 42%–47% B at 70–80 min, 47%–51% B at 80–88 min, 51%–71% B at 88–110 min. The injection volume was 20 μL, and the column temperature was set to 35°C. The mass spectrometry analysis was performed in both positive and negative ion modes in a range of 100–1100 Da. The optimized parameters of the ESI source were set as follows: drying gas (N_2_) flow rate, 10.0 L/min; drying gas temperature, 350°C; nebulizer pressure, 30 psig; fragmentor, 80, 135, 175, 225, 300, and 375 V; capillary voltage, −3,500 or 4000 V. The mass spectrometer was set in multiple reaction monitoring modes for quantification of selected ions.

### Animal Experiment

Male C57BL/6 mice (Six-week-old, 20 ± 2 g) were purchased from Hubei Center for disease Control and Prevention (Wuhan, China). Mice were adaptively housed for 1 week with 12 h light/dark cycle (55 ± 5% humidity, 23 ± 2°C) and free access to food and water. After that, mice were randomly divided into four groups (n = 9): 1) Ctrl group, fed with normal chow diet and administered with saline by gavage for 2 weeks; 2) CRF group, fed with 0.2% adenine diet (w/w) and administered with saline by gavage for 2 weeks; 3) SR group, fed with normal chow diet and administered with SR decoction (2.0 g crude SR/kg) for 2 weeks by gavage; 4) CRF + SR group, fed with 0.2% adenine diet (w/w) and administered with *SR* decoction (2.0 g/kg) by gavage for 2 weeks. Animal diet was bought from Chunzhilong Experimental Animal Co., Ltd. (Wuhan, China). During the animal experiment, the body weight, diet intake, and water drinking of mice were monitored. At the end of the experiment, fresh feces were collected from each mouse. Then, all mice were euthanized with the collection of the kidney, colon, cecum contents, and plasma. Colon length was measured, and the kidney tissues were photographed. All samples were stored at −80°C for further experiment.

For the antibiotic experiment, male C57BL/6 mice were randomly divided into four groups (n = 9): 1) Ctrl group, given distilled water for 4 weeks and then fed with normal chow diet plus saline by gavage for another 2 weeks; 2) CRF group, give distilled water for 4 weeks and then fed with 0.2% adenine diet (w/w) plus saline by gavage for another 2 weeks; 3) CRF + AB group, given antibiotic mixture (Abx, 1.0 mg/ml ampicillin, 1.0 mg/ml neomycin, 0.5 mg/ml vancomycin, and 0.5 mg/ml metronidazole in distilled water) for 4 weeks and then fed with 0.2% adenine diet (w/w) plus saline by gavage for another 2 weeks; 4) CRF + AB+ SR group, given Abx for 4 weeks and then fed with 0.2% adenine diet (w/w) plus *SR* decoction (2.0 g/kg) by gavage for another 2 weeks. Four weeks after the start of the animal experiment, mouse fecal samples were collected, and the DNA was extracted for the detection of bacterial content by RT-qPCR. At the end of the experiment, all mice were euthanized, and the tissues were collected as those mentioned above.

The animal experiments were performed according to the Animal Care and Use Committee of the animal facility at the Hubei University of Chinese Medicine.

### Creatinine and Urea Analysis

Creatinine Colorimetric Assay Kit and Urea Colorimetric Assay Kit were separately used to detect the levels of creatinine and urea in plasma according to the manufacturer’s instructions (Elabscience Biotechnology Co., Ltd., Wuhan, China).

### RNA Extraction and Real-Time Quantitative PCR

Based on the manufacturer’s protocol, total RNA of kidney and colon tissues was extracted using Trizol reagent and reversely transcribed to cDNA with a first-strand cDNA synthesis kit (Allmeek Co., Ltd., Beijing, China). The relative mRNA levels of target genes were measured by RT-qPCR using a SYBR QPCR mixture (Allmeek Co., Ltd., Beijing, China) at the ABI 7500 Real-Time Fluorescence Quantitative PCR instrument. The primer sequences were listed in Supplementary Tables S2, S3. The thermal cycle condition was as follows: pre-denaturing at 95°C for 10 min; 40 cycles of denaturation at 95°C for 10 s, annealing/extension at 60°C for 30 s. Target gene expressions were normalized against that of β-actin, and fold changes were calculated using a 2^(−ΔΔCT)^ method.

### Histological Analysis

Kidney and colon tissues were fixed with 4% paraformaldehyde, dehydrated, paraffin-embedded, and cut into 5 μm-thick sections. After the deparaffinization using xylene and seriously diluted ethanol, sections were stained with hematoxylin and eosin (H&E). Besides, Periodic Acid-Schiff (PAS) Staining Kit (Solaibao Co., Ltd., Beijing, China) and MASSON Staining Kit (Heart Biological Co., Ltd., Xian, China) were used to evaluate the levels of glomerulosclerosis and renal fibrosis, respectively. Alcian blue staining was performed to evaluate the acidic mucin expression in colon tissues following the manufacturer’s instruction (Vectorlabs, Beijing, China). Glycosylated mucin expression in colon tissues was stained using Wheat Germ Agglutinin (WGA)-FITC (Sigma, St. Louis, MO, United States). Images were acquired by a Leica DFC310 FX digital camera connected to a Leica DMI4000B light microscope (Wetzlar, Germany).

### Quantification of Intestinal Metabolites in Feces

To quantify the levels of BAs in feces, we homogenized 50 mg of fecal samples with 1 ml of water-methanol-formic acid solution (25:74:1, V/V/V) containing d_5_-CA and d_4_-TCA as internal standards at a final concentration of 0.2 μg/ml. To determine the contents of SCFAs, we homogenized 50 mg of fecal sample with 1 ml of 50% (V/V) methanol-aqueous solution (containing 0.2% HCl). To detect the level of indole, we homogenized 25 mg of fecal sample with 1 ml of pre-cooled methanol. All samples were used for GC-MS or LC-MS analysis. The detailed analytical information was indicated in Supplementary Methods.

### 16S rDNA Gene Sequencing

The total mouse fecal genome was extracted, and intestinal flora was detected by sequencing the V3-V4 region of 16S rDNA on the Illumina MiSeq platform (Illumina, San Diego, CA, United States). The metagenomic DNA from mouse colonic contents was obtained using a FastDNA™ SPIN Kit (MP Biomedicals, CA, United States). The V3-V4 variable region was amplified using barcoded primers. The PCR product was detected by 1% agarose gel electrophoresis and purified with Agencourt AMPure XP Nucleic acid purification kit. The amplicons were then pooled in paired-end sequence on an Illumina MiSeq platform (Illumina, Journal Pre-proof 9 San Diego, CA, United States) by Beijing Allwegene Tech (Beijing, China) following the standard protocols. The detailed analytical information was indicated in Supplementary Methods.

### Western Blot

Total protein was extracted from kidney tissues using RIPA buffer (Beyotime, Shanghai, China) supplemented with a protease inhibitor cocktail (Merck, Darmstadt, Germany). Then, protein concentrations were determined using a bicinchoninic acid (BCA) protein assay kit (Beyotime, Shanghai, China). Protein samples were separated on sodium dodecyl sulfate polyacrylamide gel electrophoresis (SDS-PAGE) gels and transferred to polyvinylidene difluoride (PVDF) membranes. After blocking with 5% skim milk in Tris-buffered saline tween-20 (TBST) for 1 h, the membranes were separately incubated with primary antibodies at 4°C overnight, including E-Cadherin, β-Catenin, p-GSK-3β (ser 9), GSK-3β, and β-actin. After the wash with TBST, membranes were incubated with secondary antibody conjugated with horseradish peroxidase (HRP) for 1.5 h. Finally, protein signals were visualized using an ECL Protein Detection kit.

### Statistical Analysis

Data were presented as mean ± SD. The difference between the two groups was analyzed using an unpaired two-tailed Student’s *t*-test. Differences among multiple groups were assessed using a one-way ANOVA and Bonferroni post-hoc analysis. And *p* < 0.05 was considered statistically significant. Regular analysis was carried out using GraphPad Prism (Version 8.0.1, GraphPad Software Inc., CA, United States).

## Results

### Component Identification of SR Decoction by HPLC and MS

**FIGURE 1:**
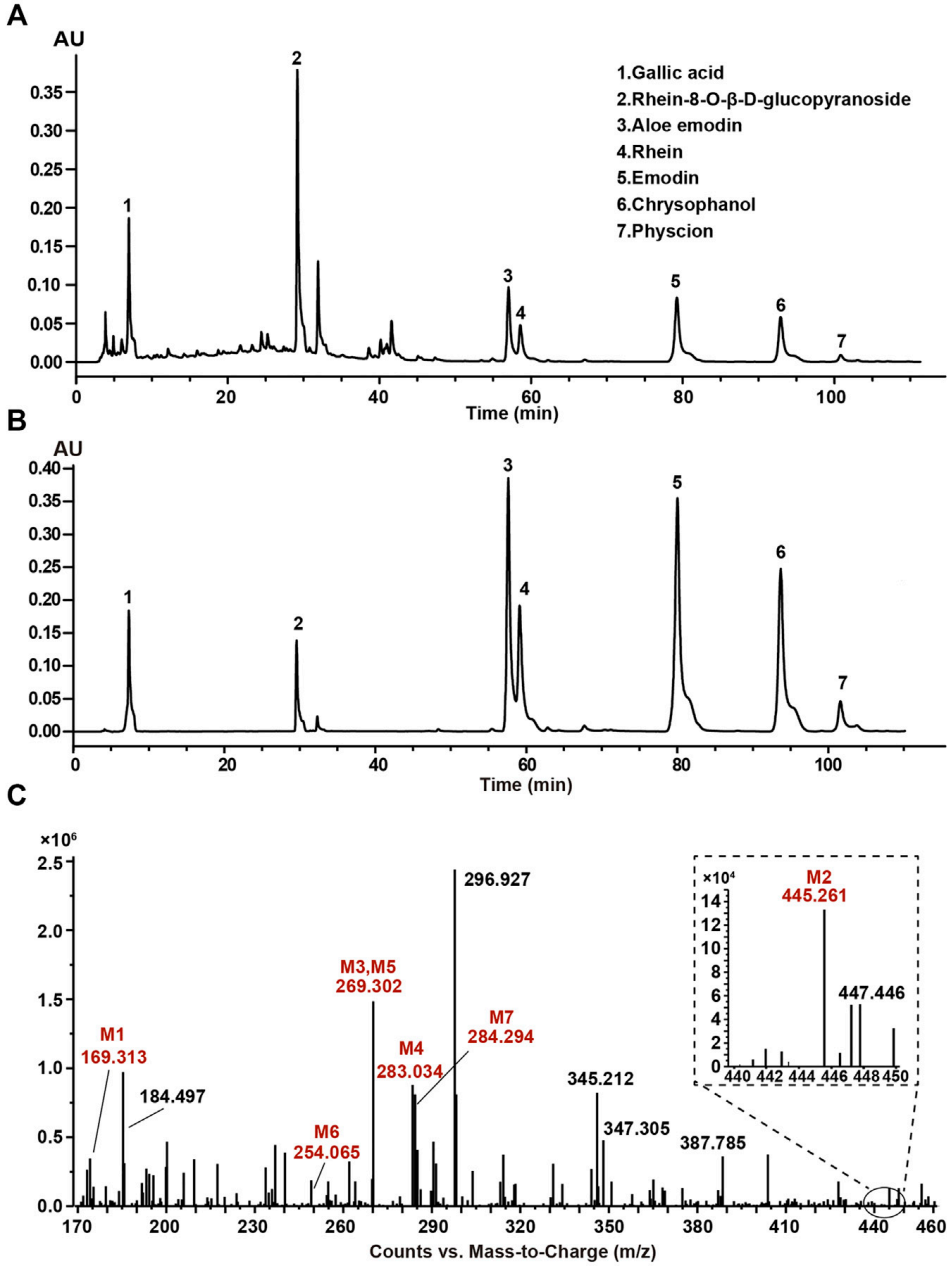
Composition analysis of SR decoction by high-performance liquid chromatography (HPLC) and mass spectrometry. **(A)** HPLC analysis of SR decoction. **(B)** HPLC analysis of related standards. Peak 1, Gallic acid; Peak 2, Rhein-8-O-β-D-glucopyranoside; Peak 3, Aloe emodin; Peak 4, Rhein; Peak 5, Emodin; Peak 6, Chrysophanol; Peak 7, Physcion. **(C)** MS spectra of SR decoction by mass spectrometry. M1, Gallic acid; M2, Rhein-8-O-β-D-glucopyranoside; M3, Aloe emodin; M4, Rhein; M5, Emodin; M6, Chrysophanol; M7, Physcion.

The typical HPLC chromatogram of SR decoction (Figure 1A) was mainly composed of seven components as compared to related standards (Figure 1B): Peak 1, Gallic acid; Peak 2, Rhein-8-O-β-D-glucopyranoside; Peak 3, Aloe emodin; Peak 4, Rhein; Peak 5, Emodin; Peak 6, Chrysophanol; Peak 7, Physcion. Further, these components were confirmed and quantified by mass spectrometry analysis, as shown in Figure 1C and Table 1, Supplementary Figure S1, and Supplementary Table S4.

**TABLE 1:** Typical MS data of major compounds in SR decoction.

Peak no	Name	Formula	Molecular mass	Measured [M-H] ˉ (m/z)	Fragment ions
M1	Gallic acid	C_7_H_6_O_5_	170	169.010	124.9
M2	Rg	C_21_H_18_O_11_	446	445.261	240.1, 284.0
M3	Aloe emodin	C_15_H_10_O_5_	270	269.302	239.2
M4	Rhein	C_15_H_8_O_6_	284	283.034	239.0
M5	Emodin	C_15_H_10_O_5_	270	269.302	225.0
M6	Chrysophanol	C_15_H_10_O_4_	254	254.093	209.7, 225.3
M7	Physcion	C_15_H_8_O_6_	284	284.294	211.7, 239.4, 268.7

### SR Decoction Improved Physiochemical Parameters of CRF Mice

**FIGURE 2:**
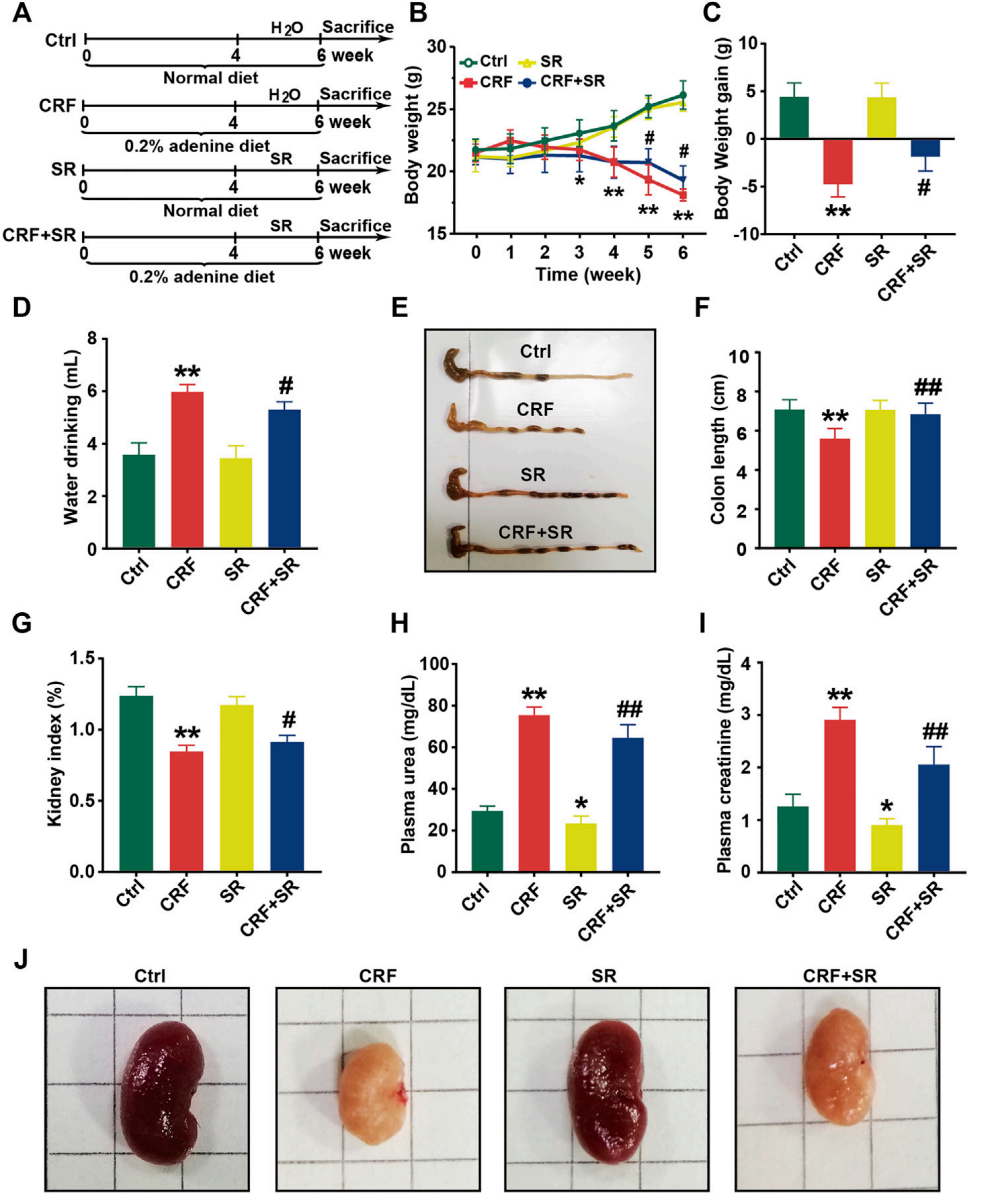
Improvement of physiochemical parameters in adenine-induced CRF mice by SR decoction. **(A)** Experimental schematic diagram. **(B)** Growth curve. **(C)** Body weight gain. **(D)** Water drinking. **(E)** Macroscopic observation of colon. **(F)** Measurement of colon length. **(G)** Kidney index. **(H)** Level of plasma urea. **(I)** Leve of plasma creatinine. **(J)** Macroscopic observation of kidney. Data were presented as mean ± SD (n = 8). ^*^ *p* < 0.05, ^**^ *p* < 0.01 *vs.* Ctrl group; ^#^ *p* < 0.05, ^##^ *p* < 0.01 *vs.* CRF group.

The schematic diagram of animal experimental was indicated in Figure 2A. In brief, mice were fed with a 0.2% adenine diet to induce CRF, and then SR decoction was used to interfere with CRF for 2 weeks. Results show that the body weight of CRF mice was gradually decreased (Figure 2B). After the treatment of SR decoction, the weight loss of CRF mice was notably inhibited (*p* < 0.05, *vs.* CRF group) (Figures 2B,C). The water drinking of CRF mice was also significantly reduced after the intervention of SR decoction (*p* < 0.05, *vs.* CRF group) (Figure 2D). As indicated in Supplementary Figure S2, the diet intake was significantly decreased in mice of the CRF group compared to the Ctrl group (*p* < 0.01), but there was no difference between the CRF group and CRF + SR group. Meanwhile, the colon length was shortened in CRF mice but corrected by SR decoction treatment (*p* < 0.05, *vs.* CRF group) (Figures 2E,F). In comparison with the CRF group, the decreased kidney index was partly reversed in the CRF + SR group (*p* < 0.05) (Figure 2G). Additionally, SR decoction suppressed the increase in levels of urea and creatinine of CRF mice (*p* < 0.05, *vs.* CRF group) (Figures 2H,I). Noticeably, SR decoction also decreased the levels of plasma urea and creatinine in the control mice (*p* < 0.05, SR group *vs.* Ctrl group) (Figures 2H,I). Finally, the kidney morphology of CRF mice was characterized by apparent shrinkage and paleness, which were improved by SR decoction treatment (Figure 2J).

### SR Decoction Suppressed Fibrosis, Inflammation, and Reduction of Aquaporins in Kidney Tissues of CRF Mice

**FIGURE 3:**
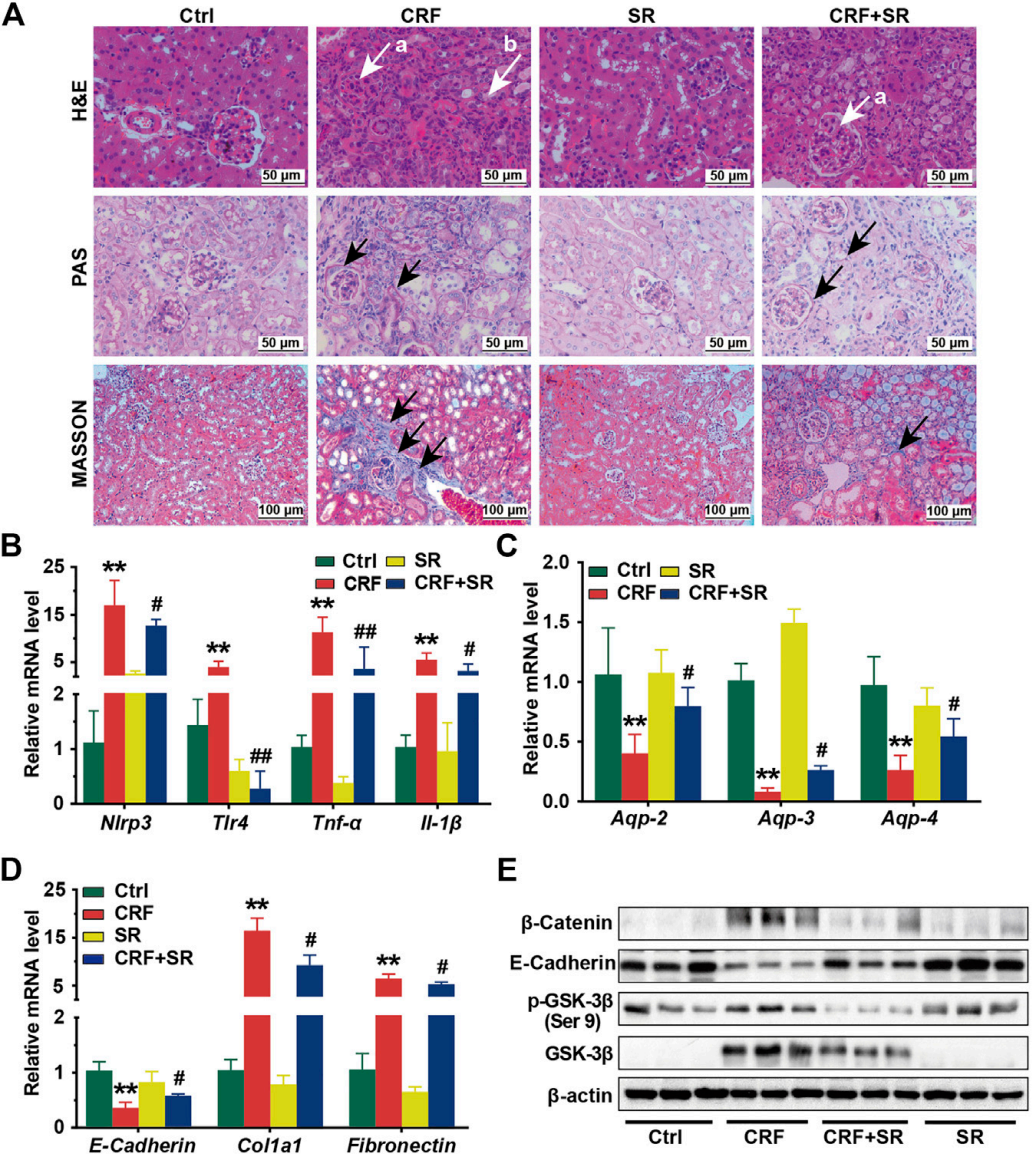
Reversal of inflammatory responses, fibrosis, reduced aquaporins in kidney tissues of CRF mice by SR decoction. **(A)** Morphological changes of kidney tissues among four experimental groups by H&E staining (400 ×, “a” indicates glomerulosclerosis and “b” indicates tubular atrophy), PAS staining (400 ×, arrows indicate lesions), and MASSON staining (200 ×, arrows indicate collagen fiber). **(B)** Expressions of proinflammatory cytokines, including *Nlrp3*, *Tlr-4*, *Tnf-α*, and *Il-1β* at mRNA levels. **(C)** Expressions of aquaporins, including *Aqp-2*, *Aqp-3*, and *Aqp-4* at mRNA levels. **(D)** Expressions of renal fibrosis-related molecules, including *E-Cadherin*, *Col1a1*, and *Fibronectin* at mRNA levels. **(E)** Expressions of renal fibrosis signaling pathway-related regulators, including β-Catenin, E-Cadherin, p-GSK-3β (ser 9), and GSK-3β at protein levels by Western blot. Data were presented as mean ± SD. ^*^ *p* < 0.05, ^**^ *p* < 0.01 *vs.* Ctrl group; ^#^ *p* < 0.05, ^##^ *p* < 0.01 *vs.* CRF group.

In Figure 3A, renal glomerular sclerosis was observed in CRF mice by HE staining, with characteristics of vacuolization and atrophy of renal tubules. Compared to the Ctrl group, PAS staining of renal tissues manifested thickening of the glomerular basement membrane and renal tubule in mice of the CRF group (Figure 3A). By MASSON staining, mice of the CRF group displayed band-like interstitial fibrosis and collagen fiber proliferation (Figure 3A). In contrast, SR decoction treatment ameliorated the above pathological changes in kidney tissues of CRF mice (Figure 3A).

To further explore the protective effect of SR decoction on kidney injuries of CRF mice, the mRNA expressions of associated regulators were analyzed by RT-qPCR. As shown in Figures 3B,C, we observed the increased expressions of proinflammatory cytokines (*Nlrp3*, *Tlr-4*, *Tnf-α*, and *Il-1β*) (*p* < 0.01, *vs.* Ctrl group) and reduced expressions of aquaporins (*Aqp-2*, *Aqp-3*, and *Aqp-4*) at mRNA levels in CRF mice kidneys (*p* < 0.01, *vs.* Ctrl group), and these changes significantly blocked by SR decoction (*p* < 0.05 or 0.01, *vs.* CRF group). Also, we detected abnormal mRNA expressions of fibrosis-related molecules in kidney tissues of CRF mice, as indicated by the down-regulation of *E-Cadherin* and up-regulation of *Col1a1* and *Fibronectin* (*p* < 0.01, *vs.* Ctrl group), which were statistically reversed by SR decoction (*p* < 0.05, *vs.* CRF group) (Figure 3D).

To gain more insight into the effect of SR decoction on the renal fibrosis signaling pathway, we examined the protein expressions of β-catenin-related transduction signals in kidneys by western blot analysis. Among these molecules, the E-Cadherin expression was reduced, but the levels of β-Catenin, p-GSK-3β (ser 9), and GSK-3β were increased in CRF mice (Figure 3E). On the contrary, SR decoction treatment remarkably inhibited the protein changes of the above regulators or kinases in CRF mice (Figure 3E).

### SR Decoction Protected Gut Barrier Against Damage in CRF Mice

**FIGURE 4:**
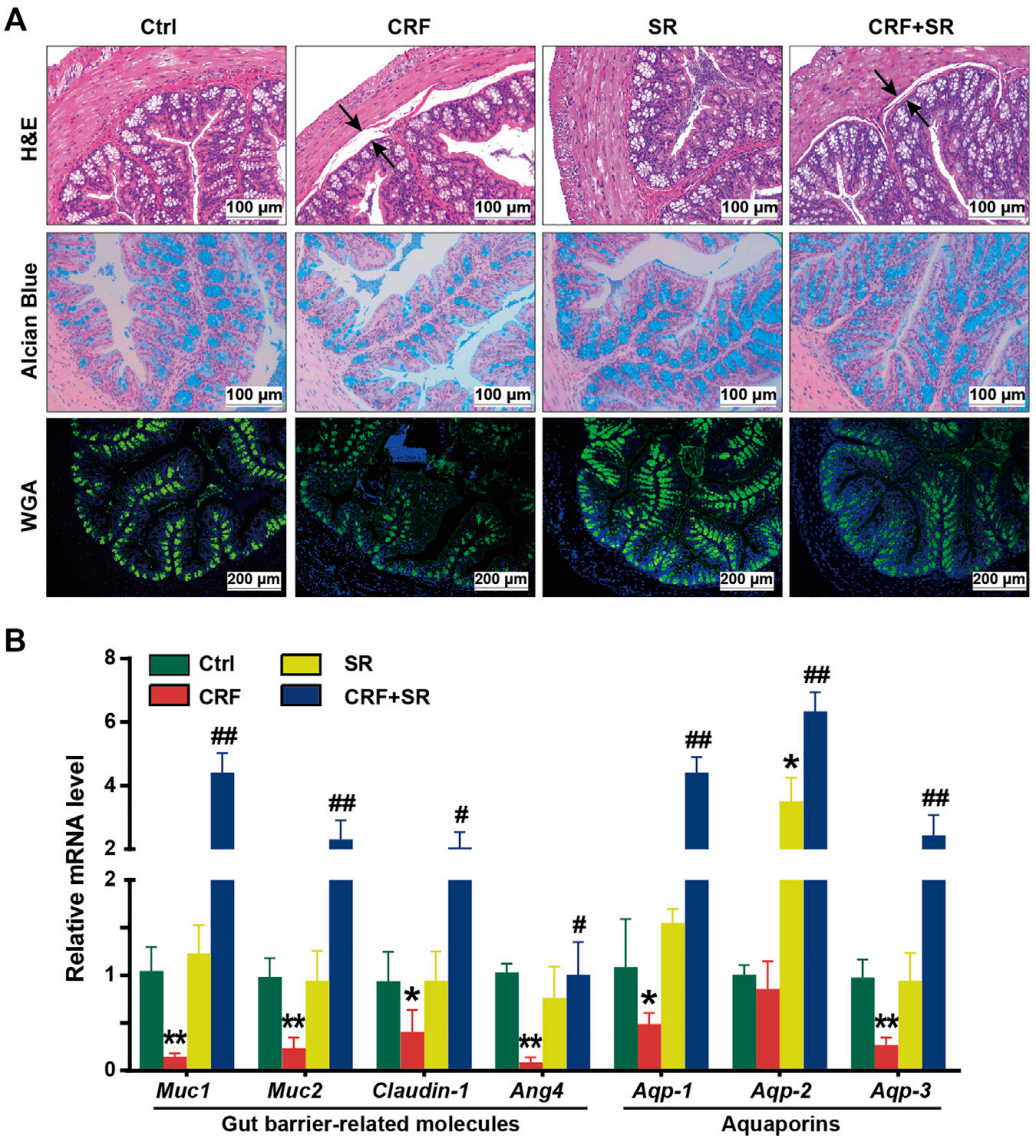
Protection of gut barrier damage in colon of CRF mice by SR decoction. **(A)** Morphology of colon tissues by using H&E staining (200 ×), Alcian blue staining (200 ×), and WGA-FITC staining (100 ×). Black arrows, indicating intestinal edema. **(B)** Expressions of gut barrier-related molecules and aquaporins in colon tissues at mRNA level by RT-qPCR. Data were presented as mean ± SD (n = 6). ^*^ *p <* 0.05, ^**^ *p* < 0.01 *vs.* Ctrl group*;* ^*#*^ *p* < 0.0*5,* ^*##*^ *p* < 0.01 *vs.* CRF group.

Since the colon length was shortened in CRF mice (Figures 2E,F), we exploited the effect of SR decoction on damage to the gut barrier of adenine diet-fed mice. As illustrated in Figure 4A, H&E staining shows an intestinal edema change between the muscular layer and mucous layers in CRF mice with thinned muscularis in the colon, and SR decoction treatment corrected these pathological changes. By WGA-FITC staining and Alcian blue staining, we found the reduced content of intestinal glycoprotein mucins in colon tissues of the CRF group, which was significantly restored by SR decoction (Figure 4A). In consistence with morphological changes, the mRNA expressions of key regulators related to the gut barrier (*Muc1*, *Muc2*, *Claudin-1*, and *Ang4*) were remarkably lowered in colon tissues of CRF mice (*p* < 0.05, *vs.* Ctrl group) but modified after SR decoction intervention (*p* < 0.05 or 0.01, *vs.* CRF group) (Figure 4B). In consideration of the intestinal edema in CRF mice, the mRNA levels of aquaporins in colon tissues were measured among experimental groups. As indicated in Figure 4B, SR decoction entirely reversed the decrease of *Aqp-1*, *Aqp-2*, and *Aqp-3* levels in CRF mice (*p* < 0.01, *vs.* CRF group).

### SR Decoction Regulated Production of Gut Microbiota Metabolites in CRF Mice

**FIGURE 5:**
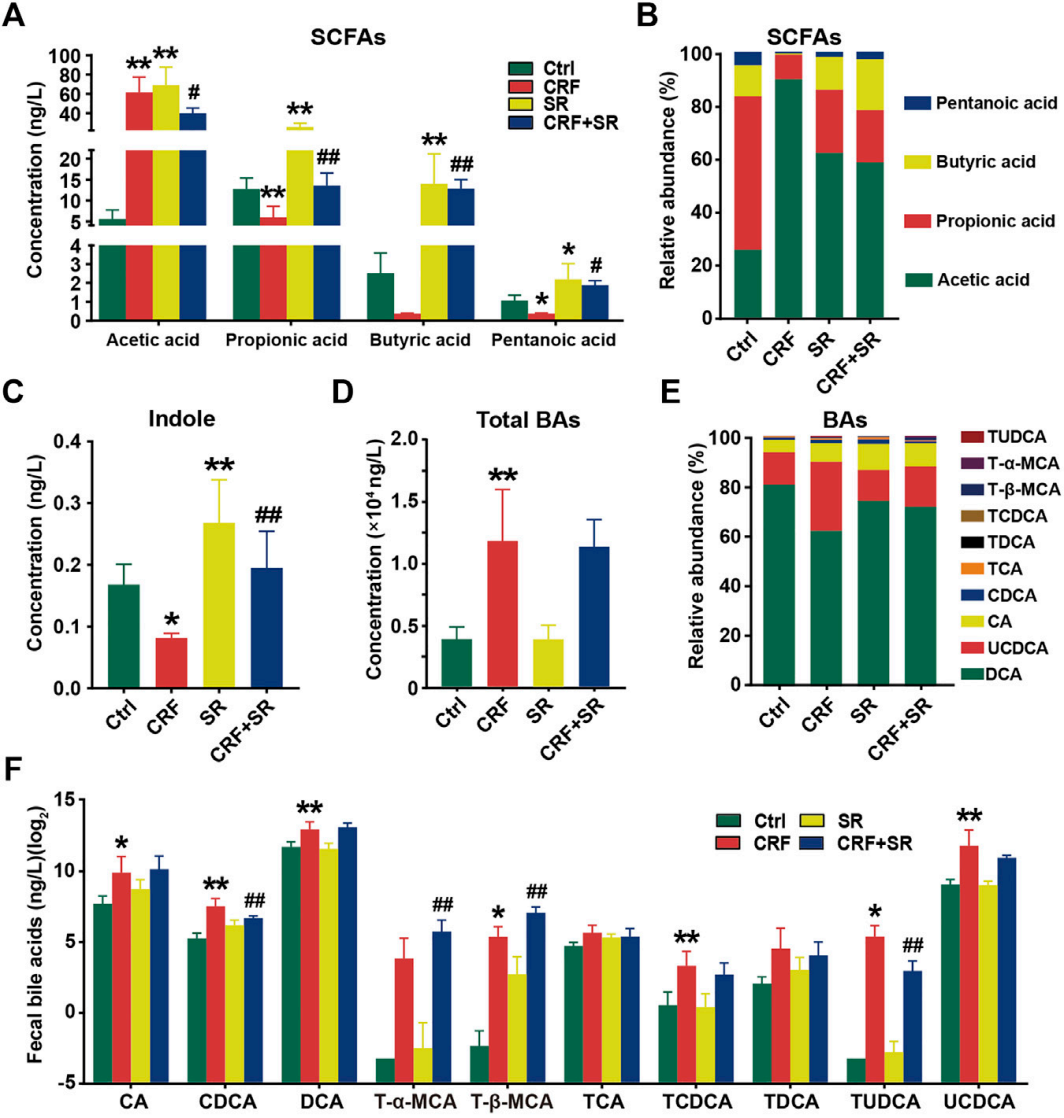
Regulatory effect of SR decoction on production of gut microbiota metabolites in CRF mice. **(A)** SCFAs contents in feces. **(B)** Relative abundances of individual SCFAs in feces. **(C)** Content of indole content in feces. **(D)** Contents of total BAs in feces. **(E)** Relative abundances of individual BAs in feces. **(F)** Quantification of individual BAs. Data were presented as mean ± SD (n = 6). ^*^ *p* < 0.05, ^**^ *p* < 0.01 *vs.* Ctrl group; ^#^ *p* < 0.05, ^##^ *p* < 0.01 *vs.* CRF group.

CRF tends to cause intestinal endotoxin accumulation, thus we quantified the contents of gut microbiota metabolites in fecal samples among four experimental groups by LC/GC-MS analysis. As shown in Figure 5A, the level of acetic acid was increased in the feces of CRF mice but remarkably decreased after *SR* decoction treatment (*p* < 0.05, *vs.* CRF group). In contrast, the fecal levels of propionic acid and valeric acid in CRF mice were reduced, whereas SR decoction statistically reversed these changes (*p* < 0.05 or 0.01, *vs.* CRF group). Notably, the contents of four SCFAs were also increased in control mice with SR decoction treatment (Figure 5A). Consistently, the relative proportions of four SCFAs in feces were changed as indicated by the increased abundance of acetic acid and decreased abundance of pentanoic acid in CRF mice, which were markedly curbed by SR decoction (Figure 5B). Meanwhile, SR decoction suppressed the reduction of fecal indole in CRF mice (*p* < 0.01, *vs.* CRF group) (Figure 5C).

Compared to the Ctrl group, the total BAs in feces were significantly increased in CRF mice (*p* < 0.01) (Figure 5D). Although SR decoction failed to block the increment of total BAs in CRF mice, it inhibited the changed proportions of several individual BAs, like UCDCA and CA (Figures 5D,E). Next, we determined the absolute contents of individual BAs in feces among experimental groups (Figure 5F). Except for T-α-MCA, TCA, and TDCA, the levels of other BAs were elevated in the feces of the CRF group (*p* < 0.05 or 0.01, *vs.* Ctrl group). Among them, the contents of TUDCA and CDCA were statistically reduced after SR decoction intervention (*p* < 0.01, *vs.* CRF group), while the contents of T-α-MCA and T-β-MCA were further promoted by SR decoction (*p* < 0.01, *vs.* CRF group) (Figure 5F).

### SR Decoction Ameliorated Gut Microbiota Dysbiosis in CRF Mice

**FIGURE 6:**
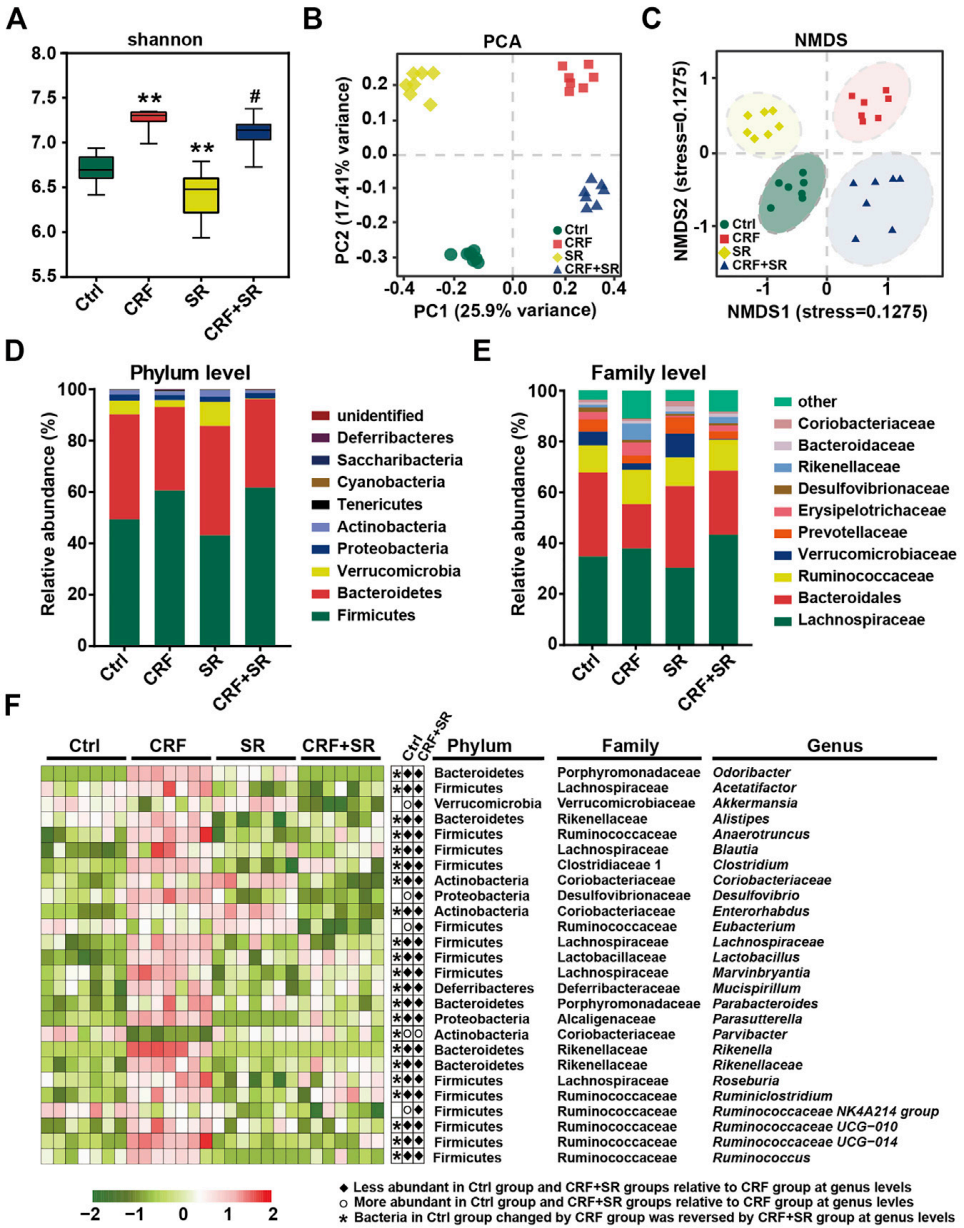
Amelioration of gut microbiota dysbiosis in CRF mice by SR decoction. **(A)** Shannon’s diversity index. **(B)** Principal component analysis (PCA). **(C)** Non-metric multidimensional scaling (NMDS). **(D)** Relative abundances at phylum levels. **(E)** Relative abundances at family levels. **(F)** Relative abundances of representative bacteria at genus levels. ^*^ *p* < 0.05, ^**^ *p* < 0.01 *vs*. Ctrl group; ^#^ *p* < 0.05, ^##^ *p* < 0.01 *vs*. CRF group.

Gut microbiota plays a vital role in producing intestinal metabolites and the maintenance of gut barrier integrity (Akchurin and Kaskel, 2015). Hence, the 16S rDNA sequencing was conducted to assay the intestinal flora in feces using an Illumina MiSeq platform. A total of 1,765,681 raw sequence reads were obtained, and 2,548 operational taxonomic units (OTUs) were yielded after the exclusion of ineligible OTUs (Supplementary Table S5). Alpha diversity was calculated by a Shannon index, which represented the richness of gut microbiota. It was shown that SR decoction reversed the up-regulation of bacterial richness in CRF mice (*p* < 0.05, *vs.* CRF group) (Figure 6A). The primary component analysis (PCA) and non-metric multidimensional scaling (NMDS) plot revealed four distinct clusters, suggesting the different structure of gut microbiota among four experimental groups (Figures 6B,C).

We performed a taxonomic analysis to quantify the relative abundances of gut microbiota among four experimental groups. At phylum levels, Firmicutes, Bacteroidetes, Verrucobacteria, Proteobacteria, and Actinobacteria were the dominant ones in fecal samples. As compared to the Ctrl group, the abundances of Firmicutes and Tenericutes phylum were up-regulated, while the contents of Verrucomicrobia and Bacteroidetes phyla were down-regulated in the CRF group (Figure 6D). SR decoction did not affect these phylum changes of CRF mice. However, it reversed the increase of Bacteroidaceae and the decrease of Rikenellaceae and Erysipelotrichaceae at family levels in the CRF group (Figure 6E). As indicated by the heat map analysis (Figure 6F), the abundances of a series of bacteria were significantly altered in the CRF group at genus levels. Among them, the content of *Parvibacter* was promoted after SR decoction treatment. In contrast, some other bacteria were decreased in abundances, including *Odoribacter*, *Acetatifactor*, *Alistipes*, *Anaerotruncus*, *Blautia, Clostridium, Desulfovibrio*, *Enterococcus, Lachnospiraceae, Marvinbryantia, Rikenellaceae*, *Rikenella*, *Ruminiclostridium*, and *Roseburia* (*p* < 0.05, *vs.* CRF group).

**FIGURE 7:**
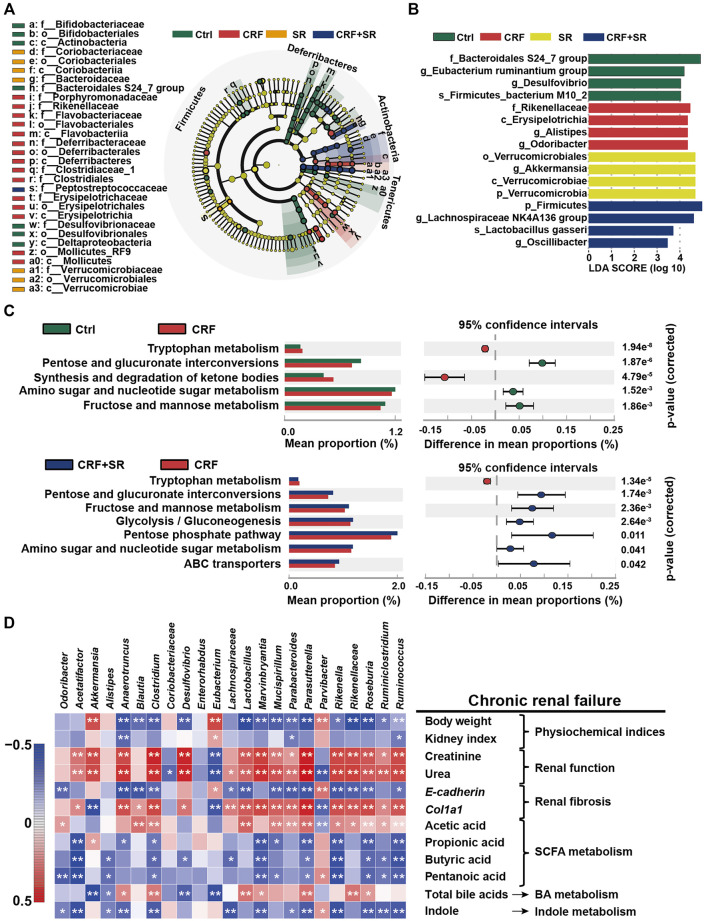
Impact of SR decoction on characteristic taxa and bacterial metabolism in CRF mice, and correlation between gut microbiota dysbiosis and SRF occurrence. **(A)** Identification of characteristic taxa among four experimental groups by linear discriminant analysis (LDA) effect size (LEfSe). **(B)** Presentation of characteristic taxa using LDA with a threshold score >3.0. Bar length of LDA represents the impact of characteristic taxa in individual groups. **(C)** Functional prediction of Kyoto Encyclopedia of Genes and Genomes (KEGG) pathways by Phylogenetic investigation of communities by reconstruction of unobserved states (PICRUSt) analysis. **(D)** Spearman’s correlation analysis between physiochemical indexes and 23 genera with the greatest changes in abundance among four experimental groups. The colors ranged from blue (negative correlation) to dark red (positive correlation), and significant correlations (n = 7) were marked by *p < 0.05, **p < 0.01.

By the LEfSe analysis, we compared the characteristic bacteria taxa among four experimental groups at distinct classification levels (“p_,” phylum; “o_,” order; “c_,” class, “f_,” family, “g_,” genus, and “s_,” species). As illustrated in Figure 7A, seven bacterial taxa were identified in the Ctrl group, which were significantly different from those of the other three experimental groups in abundance. Seven typical taxa belonged to the CRF group, and fifteen taxa were detected in the SR group. Additionally, Pptostreptococcaceae was the characteristic family in the CRF + SR group. Further, the taxa with the most remarkable differences in abundance were listed using linear discriminant analysis (LDA): 1) f_Bacteroidales S24_7 group, g_Eubacterium ruminantium group, g_Desulfovibrio, and s_Firmicutes bacterium M10_2 for the Ctrl group; 2) f_Rikenellaceae, c_Erysipelotrichia, g_Alistipes, and g_Odoribacter for the CRF group; 3) o_Verrucomicrobiales, g_Akkermansia, c_Verrucomicrobiae, and p_Verrucomicrobia for the SR group; 4) p_Firmicutes, g_Lachnospiraceae NK4A136 group, s_Lactobacillus gasseri, and g_Oscillibacter for the CRF + SR group (Figure 7B).

### SR Decoction Affected Metabolic Pathways of Intestinal Bacteria in CRF Mice

The PICRUST analysis was employed to assess the impact of SR decoction on metabolic pathways of gut microbiota in CRF mice. Based on 141 Kyoto Encyclopedia of Genes and Genomes (KEGG) pathways, seven evidently changed ones were screened for comparison among four experimental groups (Figure 7C). Compared to the Ctrl group, there were five pathways found to be affected in the CRF group (*p* < 0.01). Among them, the metabolic activity of two pathways was upregulated, including Tryptophan metabolism and synthesis/degradation of ketone bodies; three pathways were downregulated, including pentose and glucuronate interconversions, amino sugar and nucleotide sugar metabolism, and fructose and mannose metabolism. Conversely, most of these altered pathways were recovered after SR decoction treatment (*p* < 0.01 or 0.05, *vs.* CRF group) (Figure 7C).

### Correlation Between Bacterial Abundances and CRF-Related Indicators

To explore the association between gut microbiota dysbiosis and CRF occurrence, we calculated Spearman’s correlation coefficient between physiochemical indexes and 23 genera with the greatest changes in abundance among four experimental groups. As illustrated in Figure 7D, 17 bacteria (*Anaerotruncus*, *Blautia*, *Clostridium*, etc.) were negatively correlated with body weight, kidney index, E-cadherin level, and metabolism of SCFA and indole, while positively related to renal function, Col1a1, and acetic acid levels, and BA metabolism. By contrast, totally different correlations were observed between the other six genera and physiochemical parameters of CRF mice, such as *Akkermansia*, *Alistipes*, and *Coriobacteriaceae*, etc.

### SR Decoction Failed to Alleviate CRF in Mice With Gut Microbiota Depletion

**FIGURE 8:**
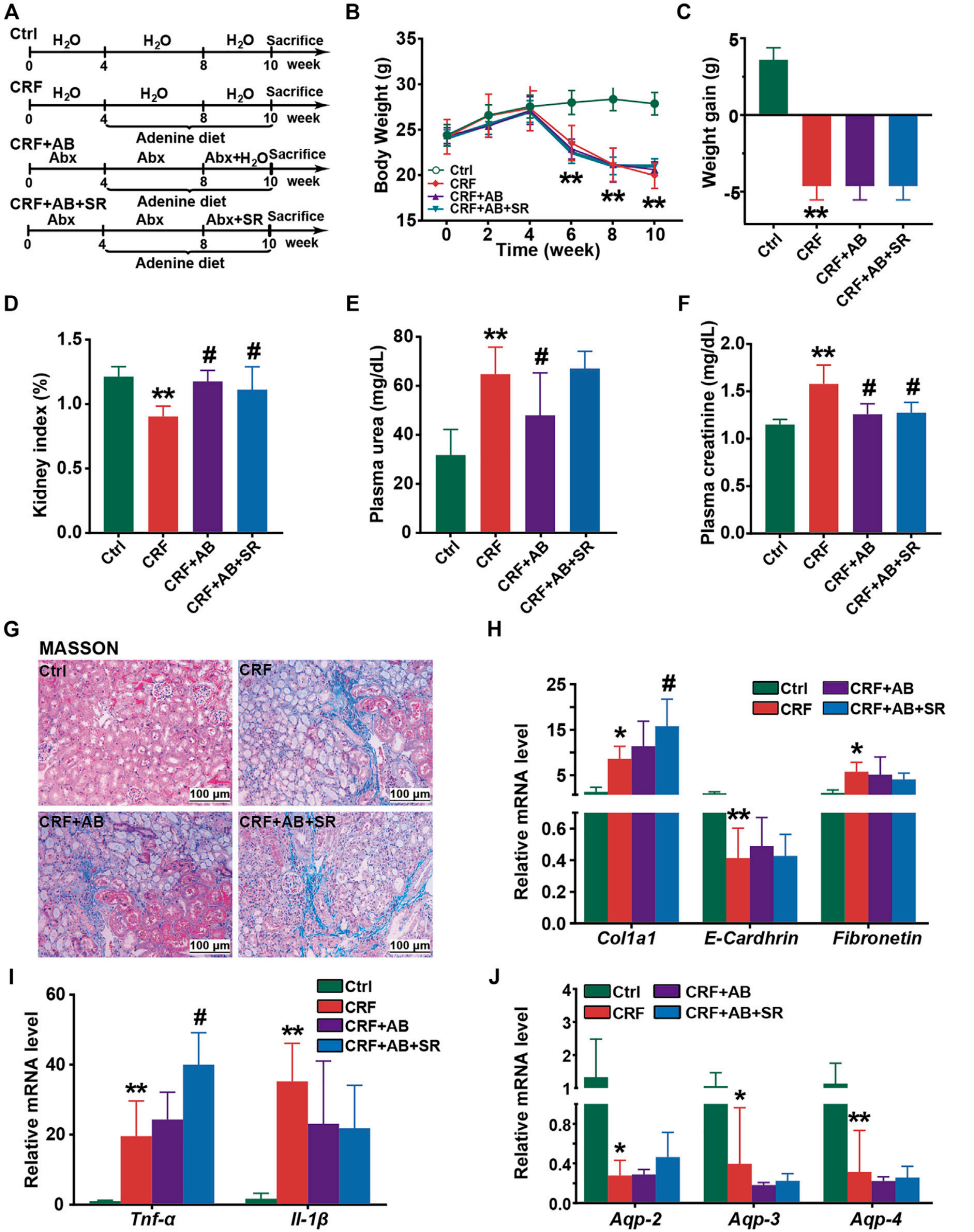
Effect of SR decoction on CRF in mice with gut microbiota depletion. **(A)** Experimental schematic diagram. **(B)** Growth curve. **(C)** Body weight gain. **(D)** Kidney index. **(E)** Level of plasma urea. **(F)** Level of plasma creatinine. **(G)** MASSON staining of kidney tissues (200 ×). **(H)** Expression of renal fibrosis biomarkers in kidney tissues at mRNA levels, including *E-Cadherin*, *Col1a1*, and *Fibronectin*. **(I)** Expression of proinflammatory cytokines in kidney tissues at mRNA levels, including *Tnf-α* and *Il-1β*. **(J)** Expression of aquaporins in kidney tissues, including *Aqp-2*, *Aqp-3*, and *Aqp-4.* Data were represented as mean ± SD (n = 6). ^*^ *p* < 0.05, ^**^ *p* < 0.01 *vs*. Ctrl group; ^#^ *p* < 0.05, ^##^ *p* < 0.01 *vs*. CRF group.

To examine whether gut microbiota played a vital role in protecting of SR decoction against CRF, we fed mice with adenine diet, adenine diet + AB, or adenine diet + AB+ SR decoction (2.0 g crude SR/kg) (Figure 8A). After AB treatment for 4 weeks, the intestinal bacteria of mice were almost depleted, as indicated by the undetectable abundances of major phyla in comparison with those of the Ctrl and CRF group (Supplementary Figure S3A). As suggested in Figure 8, most of the deteriorated physiochemical parameters in the CRF group were not improved in either the CRF + AB group or the CRF + AB+ SR group, including the growth curve (Figure 8B), weight gain (Figure 8C), renal fibrosis (Figures 8G,H) and inflammation (Figure 8I), aquaporins destruction in kidneys (Figure 8J), and other related detection indexes (Supplementary Figures S3B–S3F). As compared to the CRF group, the change of kidney index and levels of urea and creatinine in plasma were statistically suppressed in mice of the CRF + AB group due to the depletion of gut microbiota (*p* < 0.05) (Figures 8D–F). However, SR decoction treatment failed to further strengthen the AB-initiated protective effect (Figures 8D–F). Meanwhile, SR decoction also did not rescue the intestinal damage to CRF mice, as can be seen by the worsened colonic structure in the CRF + AB + SR group (Supplementary Figure S4). The above results imply that SR decoction had no alleviating effect on the occurrence of CRF in mice with gut microbiota depletion.

## Discussion

As a recognized model drug, adenine degenerates renal tubule and interstitium that causes the occurrence of CRF, characterized by the inhibited excretion of nitrogen compounds, like Creatinine and Urea (Claramunt et al., 2015). Creatinine is the metabolic end product of creatine and phosphocreatine, and urea is synthesized from ammonia in the liver (Baum et al., 1975; Jones and Brunett, 1975). Both metabolites are transported to the kidney for excretion through glomerular filtration, while impairment of the glomerulus will lead to the accumulation of toxic metabolites followed by the occurrence of CRF. In this study, we found that the levels of creatinine and urea in plasma were significantly increased in mice feeding with a 0.2% adenine diet (Figures 2H–J), accompanied by the abnormal changes of related physiochemical parameters (Figures 2, 3A). On the contrary, these typical pathological features of CRF were statistically ameliorated by SR decoction. Since there was no difference in diet intake between the CRF group and CRF + SR group, the alleviated symptoms in the CRF + SR group were not due to less adenine intake (Supplementary Figure S2). This may be attributed to an active ingredient of *Rhubarb*, i.e., Emodin. Emodin is reported to increase glomerular filtration by inhibiting glomerular podocyte apoptosis and endoplasmic reticulum stress (Tian et al., 2018).

CRF is characterized by renal fibrosis, chronic inflammation, and fluid metabolism disorder. In CRF patients, increased nitrogenous substances may contribute to systemic inflammation via the increment of pro-inflammatory cytokines, like *Nlrp3, Tlr4, Il-1β*, and *Tnf-α* (Komada and Muruve, 2019; Ebert et al., 2020). Previously, SR decoction was demonstrated to have an inhibitory effect on inflammation both *in vitro* and *in vivo* (Ye et al., 2019; Ji et al., 2020). Similarly, we found that SR decoction could suppress the activation of *Nlrp3* and decrease the mRNA levels of *Il-1β* and *Tnf-α* (Figure 3B). Aquaporin is mainly expressed in the kidney and intestine (Esteva-Font et al., 2012). Aquaporins are divided into several subtypes and play different roles in the progression of CRF. Among them, AQP-1 and AQP-3 are responsible for toxin transport like urea and ammonia, and renal tubule injury will cause decreased expressions of both aquaporins followed by delayed toxin elimination (Litman et al., 2009; Hua et al., 2019). AQP-2 not only reabsorbs water but can alleviate renal inflammation in CRF, and AQP-4 is vital for the transport of water and electrolytes (Kong et al., 2020). In the study, SR decoction significantly inhibited the downregulation of these AQPs in CRF mice, suggesting its pivotal role in the maintenance of water and toxin transport (Figure 3C). In addition, SR decoction has a therapeutic effect on renal fibrosis by partly reversing the expression changes of major biomarkers in the kidney, such as E-Cadherin, Col1a1, and Fibronectin (Figures 3D,E). E-Cadherin is an important adhesion molecule to maintain the polarity between renal tubular epithelial cells (Black et al., 2019). Col1a1 and Fibronectin can promote fibroblast differentiation, and their over-production often causes excessive fiber deposition in the kidney (Mack and Yanagita, 2015; Huang et al., 2020). In the study, SR decoction reduced the mRNA levels of *Col1a1* and *Fibronectin* in kidney tissues of CRF mice, implying its efficacy in the treatment of renal fibrosis (Figure 3D). Further, SR decoction reduced the protein levels of p-GSK-3β (ser 9), GSK-3β, and β-Catenin (Figure 3E). We presume that SR decoction ameliorated renal fibrosis dependent on a blockade of the GSK-3β/β-Catenin signaling pathway.

Notably, SR decoction displayed a protective effect on the intestinal damage of CRF mice (Figures 2, 4), and the action mechanisms may be multifactorial. On the one hand, since urea-derived ammonia and ammonium hydroxide directly damage the intestinal epithelial barrier (Huang et al., 2020), a reduced level of urea by SR decoction might benefit the gut barrier integrity in CRF mice (Figure 2H). On the other hand, the progression of CRF is accompanied by the dominance of pathogenic intestinal bacteria, leading to gut barrier corrosion (Meijers et al., 2019). Hence, the anti-bacterial effect of SR decoction makes it possible to prevent the intestine from damage (Xiang et al., 2020). Meanwhile, aquaporins play a major role in the progression of edema. The downregulation of aquaporins in the colon will deteriorate the water transport of mucosa cells and result in intestinal edema (Pelagalli et al., 2016). After SR decoction intervention, we observed significantly increased expressions of *Aqp-1*, *Aqp-2*, and *Aqp-3*, which may be the main reason for the relieved edema in colon tissues of CRF mice (Figure 4A).

As “healthy” gut microbiota products, SCFAs preferentially supply energy to intestinal epithelial cells (Yang et al., 2018). In this study, we observed the alterations of fecal SCFAs in CRF mice indicated by the increase of acetic acid and decrease of the other two SCFAs (Propionic acid and valeric acid), which were significantly repressed by SR decoction (Figure 5A). A similar result was obtained in a previous study that reported the markedly higher levels of acetic acid in feces of nephropathy mice (Yang et al., 2018). Acetic acid is involved in the citric acid cycle through the synthesis of acetyl coenzyme A and renal fibrosis will inhibit such a biological process (Hewitson and Smith, 2021), which may attribute to the sharp increase of acetic acid in CRF mice. In addition to providing the energy source for enterocytes, SCFAs have diverse regulatory functions on host physiology and immunity. For instance, SCFAs are pivotal for gut barrier integrity by promoting mucus production and suppressing inflammatory responses (Basson et al., 2016). This perhaps explains why SR decoction prevented the intestinal structure of CRF mice from damage (Figure 4). Noticeably, propionic acid can reduce urea and creatinine concentrations in the plasma of CRF patients (Huang et al., 2020), so an elevated level of propionic acid should be beneficial to the amelioration of CRF. Based on the above, the protective effect of SR decoction on CRF should be associated with its regulation of SCFA metabolic balance. Indeed, it was reported that gallic acid (a main component of SR decoction) could increase acetic acid consumption by promoting the citric acid cycle (Li et al., 2019; Wang et al., 2019).

Indole and its related bacterial metabolites were known to reduce intestinal inflammation, prevent gut barrier dysfunction, and significantly affect host metabolism (Beaumont et al., 2018). Our result shows that SR decoction statistically promoted the production of fecal indole in CRF mice (Figure 5C), suggesting its favorite effect on intestinal functions in the development of nephropathy. Aside from SCFAs and indoles, we also measured the content of fecal BAs. Of the BA pool, most BAs are taken up in the distal ileum and return to the liver. However, about 5% of the remaining BAs will escape intestinal uptake and can be metabolized by the gut microbiota to secondary BAs (Porez et al., 2012). In the intestine, BAs facilitate the digestion and absorption of dietary fat, steroids, or exogenous drug. Besides, BAs act as signaling molecules to modulate glucose and energy homeostasis (Degirolamo et al., 2014). Of note, a high concentration of BAs can cause inflammation, cellular apoptosis, or even accumulation of BAs in the liver (Kuipers et al., 2014). And patients with fatty acid diseases also have increased serum bile acids (Akchurin and Kaskel, 2015). Thus, the increase of total and individual BAs in feces may implicate the deteriorated liver function of CRF mice (Figures 5D–F). Nevertheless, SR decoction had a little or even worse effect on the alteration of fecal BAs except for CDCA and TUDCA (Figure 5F). It was suggested that BAs are not the main target of SR decoction in CRF treatment.

Considering the significance of gut microbiota in the production of intestinal metabolites, we explored the effect of SR decoction on intestinal floral structure in CRF mice. Our studies suggest that SR decoction reduced gut microbiota diversity in CRF mice (Figure 6A). This might be due to the antimicrobial effects of SR components like Rhein and Gallic acid, which were reported to inhibit the growth of some harmful bacteria, such as *Helicobacter pylori*, *Escherichia coli*, and *Streptococcus mutans* (Shao et al., 2015; Zhou et al., 2015). Though SR decoction did not affect the gut microbiota at phylum levels, it significantly regulated the abundances of several bacterial families, that is, increased the population of Bacteroidales but decreased the contents of Rikenellaceae and Erysipelotrichaceae in CRF mice (Figure 6E). Bacteroidales can alleviate renal inflammation and damage by promoting propionic and butyric acid levels (Marzocco et al., 2018). By contrast, Erysipelotrichaceae is closely related to the synthesis of phenyl sulfate, which contributes to the formation of albuminuria and the subsequent progression of CRF (Kikuchi et al., 2019). At genus levels, the abundances of a wide range of bacteria were also altered in CRF mice (Figure 6F). Among them, *Desulfovibrio* produces genotoxic hydrogen sulfide (H_2_S) gas, causing hypoplasia and hyperpermeability of intestinal epithelial cells (Rohr et al., 2020). The *Blautia*, *Acetatifactor*, and *Ruminococcus* genera are the main acetate genera, and their significant upregulation may be the reason for large increased acetic acid in CRF mouse feces (Cai et al., 2020). Genus *Parvibacter* is responsible for the metabolism of exogenous harmful substances (Choi et al., 2020). Conversely, SR decoction significantly reversed abnormal changes of these genera in CRF mice (Figure 6F). These results fully demonstrated the remodeling effect of SR decoction on the destructed structure of gut microbiota in mice with nephropathy. In a previous report, the dysbiosis of gut microbiota in CRF mice was characterized by increased pathogenic flora (Sun et al., 2021). Clinical trials also show that the abundances of Actinobacteria, Firmicutes, and Proteobacteria had the most significant increases at family levels in CRF patients compared with healthy controls (Vaziri et al., 2013). Most of the increased bacteria at genus levels also belonged to the three bacterial families in our study. Additionally, CRF can induce an increased production of urea. When the high-level urea enters the intestinal tract, it will stimulate the proliferation of bacteria with urease activity (Hobby et al., 2019). In this study, several bacteria elevated at genus levels also have urease activities, such as *Blautia*, *Ruminococcus*, *Clostridium*, *Enterorhabdus*, and *Alistipes*. The above reasons may explain why most representative bacteria genera displayed the highest levels in the CRF group (Figure 6F).

By PICRUSt analysis, SR decoction was found to affect the metabolism of gut bacteria in CRF mice, alluding to its regulatory effect on intestinal bacteria activities (Figure 7C). By correlation analysis, these altered intestinal florae were demonstrated to be associated with the production of intestinal metabolites and CRF-related physiochemical parameters (Figure 7D). Similar results were reported in clinical studies. For instance, *Eggerthella lenta* was one of the most enriched species in CRF patients and correlated with the production of several toxins (Moco et al., 2012). Further, severely aberrant gut microbiota and damaged mucosa in CRF patients displayed the potential for accelerated biosynthesis of toxic compounds, leading to a worsened kidney disease (Wang et al., 2020). It seemed that the colon mucosal barrier damage was a crucial inducer in CRF occurrence. In this study, we revealed that SR decoction had protective effects on both microbial structures and gut barrier integrity. Finally, we used germ-free mice to explore whether the original ingredients of SR decoction failed to directly protect mice against CRF before they were metabolized by intestinal flora. Indeed, the results indicate that SR decoction can’t initiate a therapeutic effect on CRF without the intestinal bacteria transformation (Figure 8). On the other hand, though antibiotic treatment partly improved CRF symptoms, plasma creatinine and urea levels were elevated in CRF mice with gut microbiota depletion compared to the Ctrl group. So did the pathological changes, such as interstitial fibrosis, collagen fiber proliferation, and renal glomerular sclerosis. These results illustrated that gut microbiota is an important but not the only factor affecting CRF formation.

Previous studies have reported the regulatory effects of *rhubarb* or its chemical components on the intestinal flora. For example, Emodin alleviated gut barrier damage in mice by improving the distribution patterns of intestinal bacteria (Zeng et al., 2016). Rhein treatment increased Lactobacillus abundance, leading to a decreased uric acid level (Wu et al., 2020). As an active ingredient in dietary polyphenols, Gallic acid could elevate the diversity of intestinal flora (Geldert et al., 2021). Besides, Rhein-8-O-β-D-glucoside will be firstly metabolized to Rhein by intestinal flora in the gut, and Rhein raised the stability of gut microbiota (Li et al., 2020). Based on the above, we speculate that several chemical compounds of SR (like Rhein, Gallic acid, and Emodin) may directly display regulatory effects on gut microbiota structure and subsequent treatment of CRF. Besides, some other components (like Rhein-8-O-β-D-glucoside and polysaccharides) will act as prodrugs, and their metabolites transformed by intestinal bacteria further eased CRF symptoms. These presumptions need to be confirmed in future work.

## Conclusion

**FIGURE 9:**
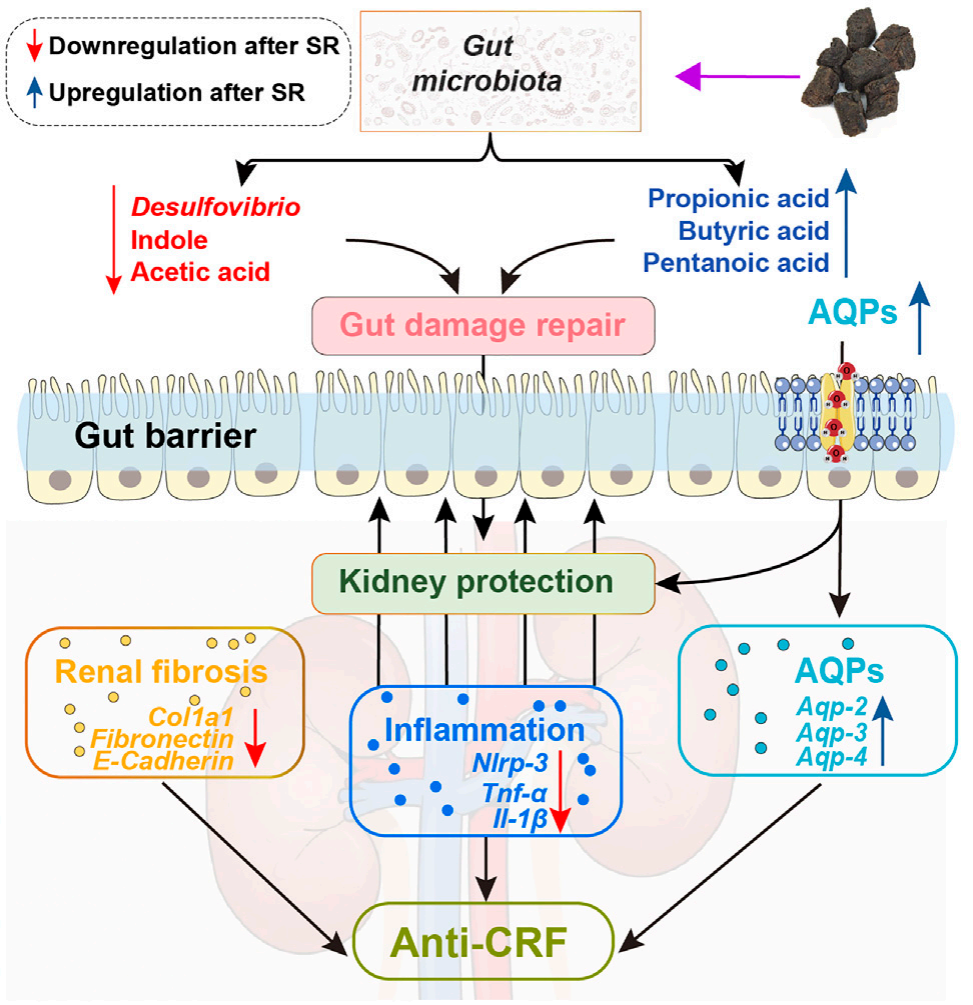
Schematic diagram showing how SR decoction improved CRF. Red arrows, decreased changes after SR decoction treatment. Blue arrows, increased changes after SR decoction treatment.

In summary, this study proved that SR decoction mitigated CRF progression in mice, as indicated by the acceleration of renal fibrosis, reversal of inflammation and abnormal water transport in the kidney, and alleviation of the deteriorated gut barrier. The potential molecular mechanisms underlying the therapeutic effects of SR decoction were related to the reshaping of imbalanced gut microbiota and suppression of abnormal intestinal metabolite production (Figure 9). These findings shed light on the potential clinical application of SR decoction in nephropathy treatment.

## Data Availability

The datasets presented in this study can be found in online repositories. The names of the repository/repositories and accession number(s) can be found below: https://www.ncbi.nlm.nih.gov/, PRJNA791187.

## Abbreviation

*SR, Stewed Rhubarb*; CRF, chronic renal failure; DHA, 2,8-dihydroxyadenine; TCM, Traditional Chinese Medicine; RT-PCR, real-time quantitative PCR; H&E, hematoxylin and eosin; PAS, Periodic acid Schiff; HPLC, High Performance Liquid Chromatography; Rg, Rhein-8-O-β-D-glucopyranoside; *Il-1β*, interleukin 1β; Tlr4, Toll-like receptor 4; *Col1a1*, Collagen alpha-1(I); *Nlrp3*, NOD-like receptor pyrin domain containing 3; *Tnf-α*, tumor necrosis factor-α; AQP, aquaporin; *Ang4*, Angiogenin 4; *Muc*, mucin; SCFAs, Short-chain fatty acids; BAs, bile acids; CA, cholic acid; TCA, taurocholic acid; CDCA, chenodeoxycholic acid; TCDCA, taurochenodeoxycholic acid; DCA, deoxycholic acid; T-α-MCA, tauro-α-murocholic acid; T-β-MCA, tauro-β-murocholic acid; TDCA, Taurodeoxycholic acid; TUDCA, taurourso-deoxycholic acid; UCDCA, Ursodeoxycholic acid; Abx, antibiotic mixture; BCA, bicinchoninic acid.

## References

[B1] Akchurin O. M. Kaskel F. (2015). Update on Inflammation in Chronic Kidney Disease. Blood Purif. 39 (1-3), 84–92. 10.1159/000368940 25662331

[B2] Ammirati A. L. (2020). Chronic Kidney Disease. Rev. Assoc. Med. Bras (1992) 66Suppl 1 (Suppl. 1), s03–s09. 10.1590/1806-9282.66.S1.3 31939529

[B3] Basson A. Trotter A. Rodriguez-Palacios A. Cominelli F. (2016). Mucosal Interactions between Genetics, Diet, and Microbiome in Inflammatory Bowel Disease. Front. Immunol. 7, 290. 10.3389/fimmu.2016.00290 27531998 PMC4970383

[B4] Baum N. Dichoso C. C. Carlton C. E. (1975). Blood Urea Nitrogen and Serum Creatinine. Physiology and Interpretations. Urology 5 (5), 583–588. 10.1016/0090-4295(75)90105-3 1093306

[B5] Beaumont M. Neyrinck A. M. Olivares M. Rodriguez J. Rocca Serra A. Roumain M. (2018). The Gut Microbiota Metabolite Indole Alleviates Liver Inflammation in Mice. FASEB j. 32, 6681–6693. 10.1096/fj.201800544 PMC6219839 29906245

[B6] Black L. M. Lever J. M. Agarwal A. (2019). Renal Inflammation and Fibrosis: A Double-Edged Sword. J. Histochem. Cytochem. 67 (9), 663–681. 10.1369/0022155419852932 31116067 PMC6713973

[B7] Cai T. T. Ye X. L. Li R. R. Chen H. Wang Y. Y. Yong H. J. (2020). Resveratrol Modulates the Gut Microbiota and Inflammation to Protect against Diabetic Nephropathy in Mice. Front. Pharmacol. 11, 1249. 10.3389/fphar.2020.01249 32973502 PMC7466761

[B8] Cao Y. J. Pu Z. J. Tang Y. P. Shen J. Chen Y. Y. Kang A. (2017). Advances in Bio-Active Constituents, Pharmacology and Clinical Applications of Rhubarb. Chin. Med. 12, 36. 10.1186/s13020-017-0158-5 29299052 PMC5745730

[B9] Chen L. Chen D. Q. Liu J. R. Zhang J. Vaziri N. D. Zhuang S. (2019). Unilateral Ureteral Obstruction Causes Gut Microbial Dysbiosis and Metabolome Disorders Contributing to Tubulointerstitial Fibrosis. Exp. Mol. Med. 51 (3), 1–18. 10.1038/s12276-019-0234-2 PMC6437207 30918245

[B10] Choi B. S. Varin T. V. St-Pierre P. Pilon G. Tremblay A. Marette A. (2020). A Polyphenol-Rich cranberry Extract Protects against Endogenous Exposure to Persistent Organic Pollutants during Weight Loss in Mice. Food Chem. Toxicol. 146, 111832. 10.1016/j.fct.2020.111832 33129933

[B11] Claramunt D. Gil-Peña H. Fuente R. García-López E. Loredo V. Hernández-Frías O. (2015). Chronic Kidney Disease Induced by Adenine: a Suitable Model of Growth Retardation in Uremia. Am. J. Physiol. Ren. Physiol 309 (1), F57–F62. 10.1152/ajprenal.00051.2015 25972508

[B12] Degirolamo C. Rainaldi S. Bovenga F. Murzilli S. Moschetta A. (2014). Microbiota Modification with Probiotics Induces Hepatic Bile Acid Synthesis via Downregulation of the Fxr-Fgf15 axis in Mice. Cell Rep 7 (1), 12–18. 10.1016/j.celrep.2014.02.032 24656817

[B13] Dos Santos I. F. Sheriff S. Amlal S. Ahmed R. P. H. Thakar C. V. Amlal H. (2019). Adenine Acts in the Kidney as a Signaling Factor and Causes Salt- and Water-Losing Nephropathy: Early Mechanism of Adenine-Induced Renal Injury. Am. J. Physiol. Ren. Physiol 316 (4), F743–f757. 10.1152/ajprenal.00142.2018 PMC6483032 30623725

[B14] Ebert T. Pawelzik S. C. Witasp A. Arefin S. Hobson S. Kublickiene K. (2020). Inflammation and Premature Ageing in Chronic Kidney Disease. Toxins (Basel) 12 (4), 227. 10.3390/toxins12040227 PMC7232447 32260373

[B15] Esteva-Font C. Ballarin J. Fernández-Llama P. (2012). Molecular Biology of Water and Salt Regulation in the Kidney. Cell Mol Life Sci 69 (5), 683–695. 10.1007/s00018-011-0858-4 21997386 PMC11114984

[B16] GBD Chronic Kidney Disease Collaboration (2020). Global, Regional, and National burden of Chronic Kidney Disease, 1990-2017: a Systematic Analysis for the Global Burden of Disease Study 2017. Lancet 395 (10225), 709–733. 10.1016/s0140-6736(20)30045-3 32061315 PMC7049905

[B17] Geldert C. Abdo Z. Stewart J. E. H S A. (2021). Dietary Supplementation with Phytochemicals Improves Diversity and Abundance of Honey Bee Gut Microbiota. J. Appl. Microbiol. 130 (5), 1705–1720. 10.1111/jam.14897 33058297

[B18] Giordano L. Mihaila S. M. Eslami Amirabadi H. Masereeuw R. (2021). Microphysiological Systems to Recapitulate the Gut-Kidney Axis. Trends Biotechnol. 39 (8), 811–823. 10.1016/j.tibtech.2020.12.001 33419585

[B19] Heinrich M. Appendino G. Efferth T. Fürst R. Izzo A. A. Kayser O. (2020). Best Practice in Research - Overcoming Common Challenges in Phytopharmacological Research. J. Ethnopharmacol. 246, 112230. 10.1016/j.jep.2019.112230 31526860

[B20] Hewitson T. D. Smith E. R. (2021). A Metabolic Reprogramming of Glycolysis and Glutamine Metabolism Is a Requisite for Renal Fibrogenesis-Why and How? Front. Physiol. 12, 645857. 10.3389/fphys.2021.645857 33815149 PMC8010236

[B21] Hobby G. P. Karaduta O. Dusio G. F. Singh M. Zybailov B. L. Arthur J. M. (2019). Chronic Kidney Disease and the Gut Microbiome. Am. J. Physiol. Ren. Physiol. 316 (6), F1211–f1217. 10.1152/ajprenal.00298.2018 PMC6620595 30864840

[B22] Hua Y. Ying X. Qian Y. Liu H. Lan Y. Xie A. (2019). Physiological and Pathological Impact of AQP1 Knockout in Mice. Biosci. Rep. 39 (5), BSR20182303. 10.1042/bsr20182303 31023968 PMC6522737

[B23] Huang Y. Zhou J. Wang S. Xiong J. Chen Y. Liu Y. (2020). Indoxyl Sulfate Induces Intestinal Barrier Injury through IRF1-DRP1 axis-mediated Mitophagy Impairment. Theranostics 10 (16), 7384–7400. 10.7150/thno.45455 32641998 PMC7330852

[B24] Ji C. Deng Y. Yang A. Lu Z. Chen Y. Liu X. (2020). Rhubarb Enema Improved Colon Mucosal Barrier Injury in 5/6 Nephrectomy Rats May Associate with Gut Microbiota Modification. Front. Pharmacol. 11, 1092. 10.3389/fphar.2020.01092 32848732 PMC7403201

[B25] Jiang S. Xie S. Lv D. Zhang Y. Deng J. Zeng L. (2016). A Reduction in the Butyrate Producing Species Roseburia Spp. And Faecalibacterium Prausnitzii Is Associated with Chronic Kidney Disease Progression. Antonie Van Leeuwenhoek 109 (10), 1389–1396. 10.1007/s10482-016-0737-y 27431681

[B26] Jones J. D. Brunett P. C. (1975). Creatinine Metabolism and Toxicity. Kidney Int. Suppl. (3), 294–298. 1057701

[B27] Kikuchi K. Saigusa D. Kanemitsu Y. Matsumoto Y. Thanai P. Suzuki N. (2019). Gut Microbiome-Derived Phenyl Sulfate Contributes to Albuminuria in Diabetic Kidney Disease. Nat. Commun. 10 (1), 1835. 10.1038/s41467-019-09735-4 31015435 PMC6478834

[B28] Koizumi M. Tatebe J. Watanabe I. Yamazaki J. Ikeda T. Morita T. (2014). Aryl Hydrocarbon Receptor Mediates Indoxyl Sulfate-Induced Cellular Senescence in Human Umbilical Vein Endothelial Cells. J. Atheroscler. Thromb. 21 (9), 904–916. 10.5551/jat.23663 24727683

[B29] Komada T. Muruve D. A. (2019). The Role of Inflammasomes in Kidney Disease. Nat. Rev. Nephrol. 15 (8), 501–520. 10.1038/s41581-019-0158-z 31164720

[B30] Kong Y. Feng W. Zhao X. Zhang P. Li S. Li Z. (2020). Statins Ameliorate Cholesterol-Induced Inflammation and Improve AQP2 Expression by Inhibiting NLRP3 Activation in the Kidney. Theranostics 10 (23), 10415–10433. 10.7150/thno.49603 32929357 PMC7482822

[B31] Kuipers F. Bloks V. W. Groen A. K. (2014). Beyond Intestinal Soap-Bbile Acids in Metabolic Control. Nat. Rev. Endocrinol. 10 (8), 488–498. 10.1038/nrendo.2014.60 24821328

[B32] Li L. Z. Tao S. B. Ma L. Fu P. (2019). Roles of Short-Chain Fatty Acids in Kidney Diseases. Chin. Med. J. (Engl) 132 (10), 1228–1232. 10.1097/cm9.0000000000000228 30946066 PMC6511413

[B33] Li Q. Guo Y. Yu X. Liu W. Zhou L. (2020). Protective Mechanism of Rhubarb Anthraquinone Glycosides in Rats with Cerebral Ischaemia-Reperfusion Injury: Interactions between Medicine and Intestinal Flora. Chin. Med. 15, 60. 10.1186/s13020-020-00341-x 32518585 PMC7275394

[B34] Li H. Feng Y. Sun W. Kong Y. Jia L. (2021). Antioxidation, Anti-inflammation and Anti-fibrosis Effect of Phosphorylated Polysaccharides from Pleurotus Djamor Mycelia on Adenine-Induced Chronic Renal Failure Mice. Int. J. Biol. Macromol 170, 652–663. 10.1016/j.ijbiomac.2020.12.159 33359803

[B35] Litman T. Søgaard R. Zeuthen T. (2009). Ammonia and Urea Permeability of Mammalian Aquaporins. Handb Exp. Pharmacol. 190, 327–358. 10.1007/978-3-540-79885-9_17 19096786

[B36] Liu H. Zheng J. Lai H. C. Hu B. Zhu L. Leung E. L. (2020). Microbiome Technology Empowers the Development of Traditional Chinese Medicine. Sci. China Life Sci. 63 (11), 1759–1761. 10.1007/s11427-020-1778-7 32789726

[B37] Ma T. T. Meng X. M. (2019). TGF-β/Smad and Renal Fibrosis. Adv. Exp. Med. Biol. 1165, 347–364. 10.1007/978-981-13-8871-2_16 31399973

[B38] Mack M. Yanagita M. (2015). Origin of Myofibroblasts and Cellular Events Triggering Fibrosis. Kidney Int. 87 (2), 297–307. 10.1038/ki.2014.287 25162398

[B39] Marzocco S. Fazeli G. Di Micco L. Autore G. Adesso S. Dal Piaz F. (2018). Supplementation of Short-Chain Fatty Acid, Sodium Propionate, in Patients on Maintenance Hemodialysis: Beneficial Effects on Inflammatory Parameters and Gut-Derived Uremic Toxins, A Pilot Study (PLAN Study). J. Clin. Med. 7 (10), 315. 10.3390/jcm7100315 PMC6210519 30274359

[B40] Meijers B. Evenepoel P. Anders H. J. (2019). Intestinal Microbiome and Fitness in Kidney Disease. Nat. Rev. Nephrol. 15 (9), 531–545. 10.1038/s41581-019-0172-1 31243394

[B41] Mishima E. Fukuda S. Shima H. Hirayama A. Akiyama Y. Takeuchi Y. (2015). Alteration of the Intestinal Environment by Lubiprostone Is Associated with Amelioration of Adenine-Induced CKD. J. Am. Soc. Nephrol. 26 (8), 1787–1794. 10.1681/asn.2014060530 25525179 PMC4520171

[B42] Miyazaki-Anzai S. Masuda M. Shiozaki Y. Keenan A. L. Chonchol M. Kremoser C. (2021). Free Deoxycholic Acid Exacerbates Vascular Calcification in CKD through ER Stress-Mediated ATF4 Activation. Kidney360 2 (5), 857–868. 10.34067/kid.0007502020 34423309 PMC8378801

[B43] Moco S. Martin F. P. Rezzi S. (2012). Metabolomics View on Gut Microbiome Modulation by Polyphenol-Rich Foods. J. Proteome Res. 11 (10), 4781–4790. 10.1021/pr300581s 22905879

[B44] Pelagalli A. Squillacioti C. Mirabella N. Meli R. (2016). Aquaporins in Health and Disease: An Overview Focusing on the Gut of Different Species. Int. J. Mol. Sci. 17 (8), 1213. 10.3390/ijms17081213 PMC5000611 27472320

[B45] Porez G. Prawitt J. Gross B. Staels B. (2012). Bile Acid Receptors as Targets for the Treatment of Dyslipidemia and Cardiovascular Disease. J. Lipid Res. 53 (9), 1723–1737. 10.1194/jlr.R024794 22550135 PMC3413216

[B46] Rohr M. W. Narasimhulu C. A. Rudeski-Rohr T. A. Parthasarathy S. (2020). Negative Effects of a High-Fat Diet on Intestinal Permeability: A Review. Adv. Nutr. 11 (1), 77–91. 10.1093/advances/nmz061 31268137 PMC7442371

[B47] Schunk S. J. Floege J. Fliser D. Speer T. (2021). WNT-β-catenin Signalling - a Versatile Player in Kidney Injury and Repair. Nat. Rev. Nephrol. 17 (3), 172–184. 10.1038/s41581-020-00343-w 32989282

[B48] Shao D. Li J. Li J. Tang R. Liu L. Shi J. (2015). Inhibition of Gallic Acid on the Growth and Biofilm Formation of *Escherichia coli* and Streptococcus Mutans. J. Food Sci. 80 (6), M1299–M1305. 10.1111/1750-3841.12902 25974286

[B49] Sun Y. B. Qu X. Caruana G. Li J. (2016). The Origin of Renal Fibroblasts/myofibroblasts and the Signals that Trigger Fibrosis. Differentiation 92 (3), 102–107. 10.1016/j.diff.2016.05.008 27262400

[B50] Sun C. Y. Li J. R. Wang Y. Y. Lin S. Y. Ou Y. C. Lin C. J. (2021). Indoxyl Sulfate Caused Behavioral Abnormality and Neurodegeneration in Mice with Unilateral Nephrectomy. Aging (Albany NY) 13 (5), 6681–6701. 10.18632/aging.202523 33621199 PMC7993681

[B51] Tian N. Gao Y. Wang X. Wu X. Zou D. Zhu Z. (2018). Emodin Mitigates Podocytes Apoptosis Induced by Endoplasmic Reticulum Stress through the Inhibition of the PERK Pathway in Diabetic Nephropathy. Drug Des. Devel Ther. 12, 2195–2211. 10.2147/dddt.S167405 PMC6047613 30034224

[B52] Vaziri N. D. Wong J. Pahl M. Piceno Y. M. Yuan J. DeSantis T. Z. (2013). Chronic Kidney Disease Alters Intestinal Microbial flora. Kidney Int. 83 (2), 308–315. 10.1038/ki.2012.345 22992469

[B53] Wang V. Vilme H. Maciejewski M. L. Boulware L. E. (2016). The Economic Burden of Chronic Kidney Disease and End-Stage Renal Disease. Semin. Nephrol. 36 (4), 319–330. 10.1016/j.semnephrol.2016.05.008 27475662

[B54] Wang S. Lv D. Jiang S. Jiang J. Liang M. Hou F. (2019). Quantitative Reduction in Short-Chain Fatty Acids, Especially Butyrate, Contributes to the Progression of Chronic Kidney Disease. Clin. Sci. (Lond) 133 (17), 1857–1870. 10.1042/cs20190171 31467135

[B55] Wang X. Yang S. Li S. Zhao L. Hao Y. Qin J. (2020). Aberrant Gut Microbiota Alters Host Metabolome and Impacts Renal Failure in Humans and Rodents. Gut 69 (12), 2131–2142. 10.1136/gutjnl-2019-319766 32241904 PMC7677483

[B56] Wu J. Wei Z. Cheng P. Qian C. Xu F. Yang Y. (2020). Rhein Modulates Host Purine Metabolism in Intestine through Gut Microbiota and Ameliorates Experimental Colitis. Theranostics 10 (23), 10665–10679. 10.7150/thno.43528 32929373 PMC7482825

[B57] Wyngaarden J. B. Dunn J. T. (1957). 8-Hydroxyadenine as the Intermediate in the Oxidation of Adenine to 2, 8-dihydroxyadenine by Xanthine Oxidase. Arch. Biochem. Biophys. 70 (1), 150–156. 10.1016/0003-9861(57)90088-7 13445250

[B58] Xiang H. Zuo J. Guo F. Dong D. (2020). What We Already Know about Rhubarb: a Comprehensive Review. Chin. Med. 15, 88. 10.1186/s13020-020-00370-6 32863857 PMC7448319

[B59] Yang J. Li Q. Henning S. M. Zhong J. Hsu M. Lee R. (2018). Effects of Prebiotic Fiber Xylooligosaccharide in Adenine-Induced Nephropathy in Mice. Mol. Nutr. Food Res. 62, 1800014. 10.1002/mnfr.201800014 29676858

[B60] Yao M. Gao J. Li G. Q. Xie Z. (2012). Quantifying Four-Probe Metabolites in a Single UPLC-MS/MS Run to Explore the Effects of Cooked Rhubarb on Cytochrome P450 Isozymes. Bioanalysis 4 (22), 2693–2703. 10.4155/bio.12.236 23210652

[B61] Ye B. Chen X. Dai S. Han J. Liang X. Lin S. (2019). Emodin Alleviates Myocardial Ischemia/reperfusion Injury by Inhibiting Gasdermin D-Mediated Pyroptosis in Cardiomyocytes. Drug Des. Devel Ther. 13, 975–990. 10.2147/dddt.S195412 PMC6438141 30988600

[B62] Zeng Y. Q. Dai Z. Lu F. Lu Z. Liu X. Chen C. (2016). Emodin via Colonic Irrigation Modulates Gut Microbiota and Reduces Uremic Toxins in Rats with Chronic Kidney Disease. Oncotarget 7 (14), 17468–17478. 10.18632/oncotarget.8160 27003359 PMC4951226

[B63] Zhang Q. Liu L. Lin W. Yin S. Duan A. Liu Z. (2017). Rhein Reverses Klotho Repression via Promoter Demethylation and Protects against Kidney and Bone Injuries in Mice with Chronic Kidney Disease. Kidney Int. 91 (1), 144–156. 10.1016/j.kint.2016.07.040 27692562

[B64] Zhang Z. H. Li M. H. Liu D. Chen H. Chen D. Q. Tan N. H. (2018). Rhubarb Protect against Tubulointerstitial Fibrosis by Inhibiting TGF-β/Smad Pathway and Improving Abnormal Metabolome in Chronic Kidney Disease. Front. Pharmacol. 9, 1029. 10.3389/fphar.2018.01029 30271345 PMC6146043

[B65] Zhou Y. X. Xia W. Yue W. Peng C. Rahman K. Zhang H. (2015). Rhein: A Review of Pharmacological Activities. Evid. Based Complement. Alternat Med. 2015, 578107. 10.1155/2015/578107 26185519 PMC4491579

[B66] Zhou S. He Y. Zhang W. Xiong Y. Jiang L. Wang J. (2021). Ophiocordyceps Lanpingensis Polysaccharides Alleviate Chronic Kidney Disease through MAPK/NF-κB Pathway. J. Ethnopharmacol. 276, 114189. 10.1016/j.jep.2021.114189 33964361

[B67] Zhu T. Liu X. Wang X. Cao G. Qin K. Pei K. (2016). Profiling and Analysis of Multiple Compounds in Rhubarb Decoction after Processing by Wine Steaming Using UHPLC-Q-TOF-MS Coupled with Multiple Statistical Strategies. J. Sep. Sci. 39 (15), 3081–3090. 10.1002/jssc.201600256 27291339

[B68] Zhuang T. Gu X. Zhou N. Ding L. Yang L. Zhou M. (2020). Hepatoprotection and Hepatotoxicity of Chinese Herb Rhubarb (Dahuang): How to Properly Control the "General (Jiang Jun)" in Chinese Medical Herb. Biomed. Pharmacother. 127, 110224. 10.1016/j.biopha.2020.110224 32559851

