# Supplementary material for: Stewed Rhubarb Decoction Ameliorates Adenine-Induced Chronic Renal Failure in Mice by Regulating Gut Microbiota Dysbiosis
Source: Front Pharmacol. 2022 Mar 21;13:842720. doi: 10.3389/fphar.2022.842720 (PMC8979777; doi:10.3389/fphar.2022.842720)
Supplement: Supplementary file 1 [file DataSheet1.docx]

Supplementary Material

# Supplementary Data

# 1.1 16S rDNA gene sequencing

# The metagenomic DNA from mouse colonic contents was obtained using a FastDNA™ SPIN Kit (MP Biomedicals, CA, USA). The V3-V4 variable region was amplified using barcoded primers (Forward: 5′-ACTCCTACGGGAGGCAGCAG-3′ and Reverse: 5′-GACTACHVGGGTWTCTAAT-3′). PCR amplification was performed as followed: initiation at 95 °C for 5 min, 20 cycles of (95 °C for 30 s, 55 °C for 30 s, and 72 °C for 30 s), and a final extension of 72 °C for 10 min. The amplicons were then purified and pooled in paired-end sequence on an Illumina MiSeq platform (Illumina, Journal Pre-proof 9 San Diego, CA, USA) by Beijing Allwegene Tech (Beijing, China) following the standard protocols. After the sequencing, data were processed by QIIME toolkit (Quantitative Insights Into Microbial Ecology, USA) subsequently. The high-quality reads with similarity > 97% were defined as an operational taxonomic unit (OTU) using UCLUST (Version 1.2.22 http://www.drive5.com/uclust/ downloads1_2_22q.html), while sequences shorter than 110 nucleotides or sequences with overlap less than 10 bp were removed. Then chimeric sequences were screened and discarded using Usearch (Version 8.1.1861 http://www.drive5. com/usearch/). The taxonomy of each 16S rDNA gene sequence was analyzed by UCLUST against the Silva 16S rRNA database (Release119 http://www.arb-silva.de) using a confident threshold of 90%. Finally, an OTU table was produced for abundance information and further analysis. The Linear Discriminant Analysis Effect Size (LEfSe) analysis was used to determine the significantly important microbial group as previously reported. The functional profile of the bacterial community data set was explored by a database of phylogenetically referenced genomes (PICRUSt, Phylogenetic Investigation of Communities by Reconstruction of Unobserved States).

# 1.2 Liquid Chromatography-Mass Spectrometry (LC-MS) analysis on Bile acids (BAs)

# BAs were analyzed by Agilent 6460 Triple Quad Mass Spectrometer with Agilent 1260 HPLC (Agilent, CA, USA) equipped with a Phenomenex Kinetex C18 column (150 × 2.1 mm × 2.6 μm) (Phenomenex, CA, USA). The mobile phase consisted of both 5 mM ammonium formate solution (A) and 70% acetonitrile plus 30% methanol (B). The gradient elution program was conducted as follows: 0‒3 min, 10% to 25% B; 3‒7 min, 25% to 30% B; 7‒11 min, 30% to 50% B; 11‒12 min, 50% to 70% B; 12‒17 min, 70% to 100% B; 17‒18 min, 100% B; 18.01‒22 min, 10% B. The volume of injection was 10 μL and the flow rate of mobile phase was 0.45 mL/min. The electrospray ionization source in negative ion pattern was used in MS analysis, accompanied by the following parameters: capillary voltage, 3.5 kV; cone hole voltage, 40 V; ion source compensation voltage, 80 V; temperature and flow rate of drying gas, 400 °C and 800 L/h. Cholic acid (CA), deoxycholic acid (DCA), taurocholic acid (TCA), ursodeoxycholic acid (UDCA), taurodeoxycholic acid (TDCA), taurochenodeoxycholic acid (TCDCA), tauroursodeoxycholic acid (TUDCA), tauro-α-murocholicacid (T-α-MCA) and tauro-β-murocholicacid (T-β-MCA) were used as internal standards.

# 1.3 Gas Chromatography-Mass Spectrometry (GC-MS) analysis on SCFAs and indole

# SCFAs and indole were detected by Agilent GC/MS 5975 (Agilent, CA, USA) equipped with a DB-WAX column (30 m × 0.32 mm × 0.5 μm) (Agilent, CA, USA). The oven temperature of the column was programmed as following heating procedure: the initial temperature was kept at 50 °C for 1 min, then raised 10 °C/min to 250 °C and maintained for 2 min with high purity helium (purity > 99.999 %) as carrier gas (flow rate: 1.0 mL/min). Fecal samples were injected at a diffluent rate of 1:50 accompanied by an electron impact source. The electron energy was 70 eV. The ion source temperature and interface temperature were set as 230 °C and 250 °C, respectively. The solvent delay time was set to 6 min. Acetic acid, propinoic acid, butyric acid, valeric acid, and indole were used as standard references.

# 1.4 Extraction and quantification of fecal bacterial genomic DNA

# Twenty-five mg of mouse feces was homogenized with 1% sterile PBS until the feces became mushy. The fecal suspension was centrifuged at ×g for 5 min. The obtained bacterial solution was treated with proteinase K, RNase, and lysozyme in a shaker at 37 °C for 1 h. Then, the lysate and DNA extraction agent were added to the bacterial solution and placed in a 65 °C water bath for 10 min, followed by the 13,000 ×g centrifugation for 10 min. Next, the supernatant was collected for the precipitation of DNA using isopropyl alcohol. After the centrifugation, the precipitated DNA was washed with 75% ethanol and dissolved in sterile water. Fecal bacterial genomic DNA was quantified on the CFX Connect Real-time system (Bio-Rad, Hercules, CA, USA) by using 2 × FastHS SYBR QPCR mixture (AllMEEK, Beijing, China). The primer sequences of All bacteria, Bacteroides, Bifidobacterium, and Salmonella were provided in Supplementary Table 3. The plasmids (Bacteroides-T, Bifidobacterium-T, and Salmonella-T) were used as the standard bacteria, and the bacterial copy number was calculated using the absolute quantification method.

# Supplementary Figures and Tables

## Supplementary Figures


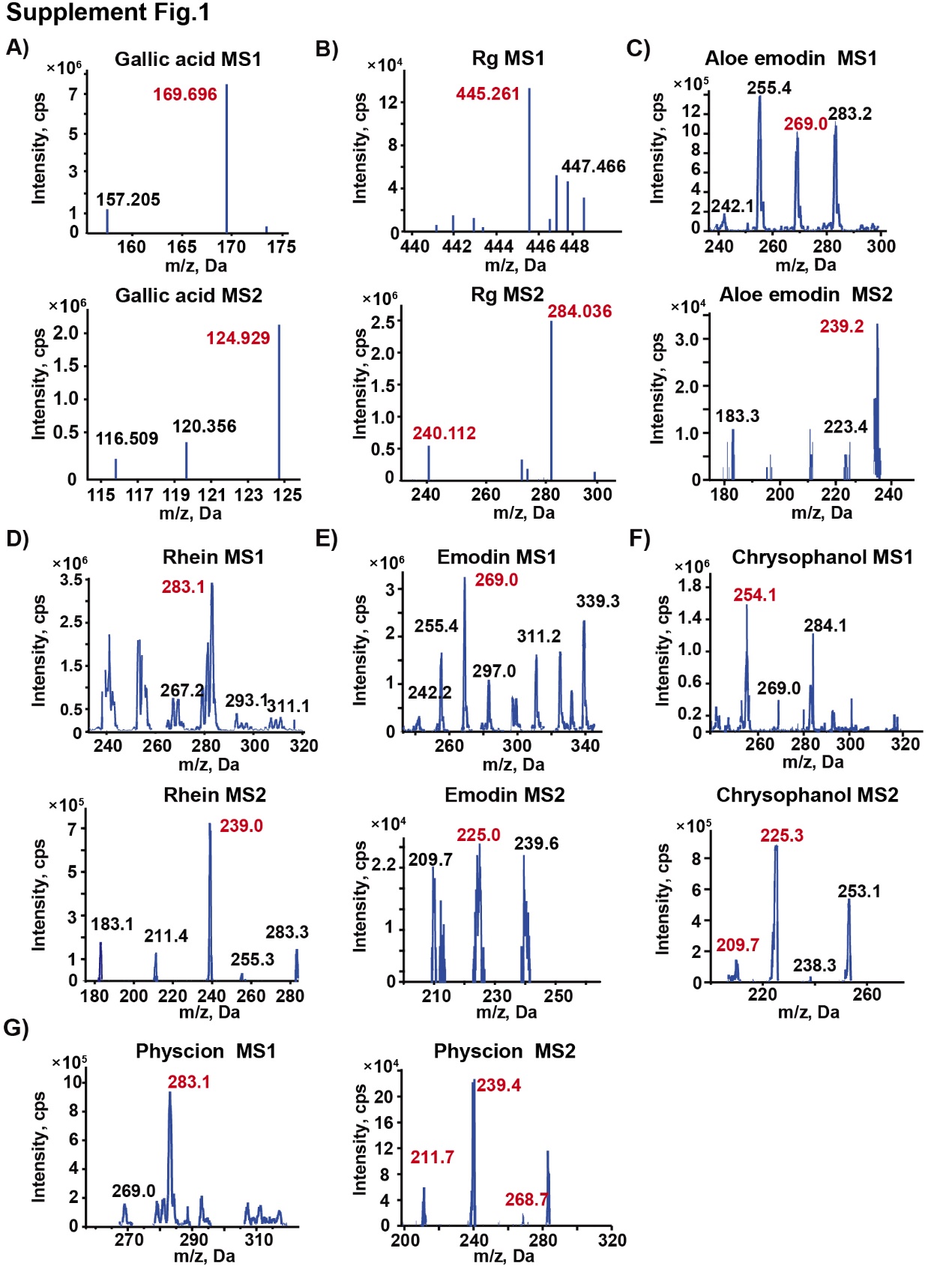


**Supplementary Figure 1.** Component identification of SR decoction by HPLC-MS/MS. (A) Mass spectra of Gallic acid. (B) Mass spectra of Rhein-8-O-β-D-glucopyranoside (Rg). (C) Mass spectra of Aloe emodin. (D) Mass spectra of Rhein. (E) Mass spectra of Emodin. (F) Mass spectra of Chrysophanol. (G) Mass spectra of Physcion.


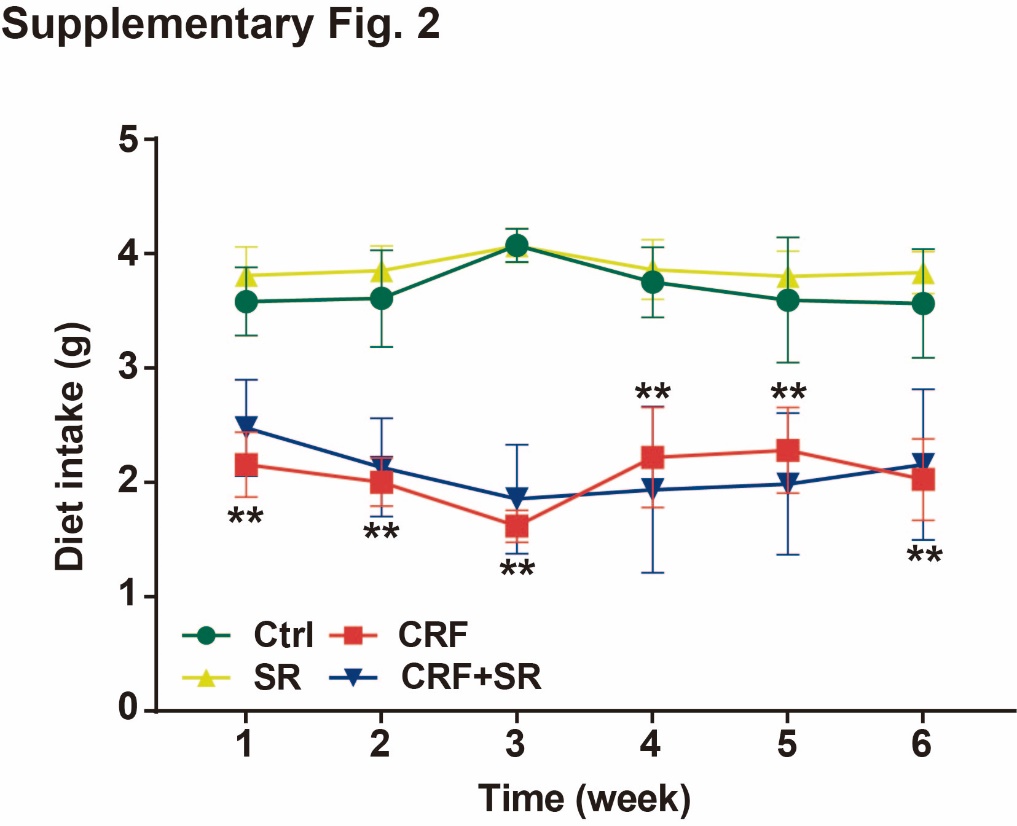


**Supplementary Figure 2.** Diet intake among four experimental groups. Data were presented as mean ± SD (n = 8). ***P* < 0.01 *vs.* Ctrl group.


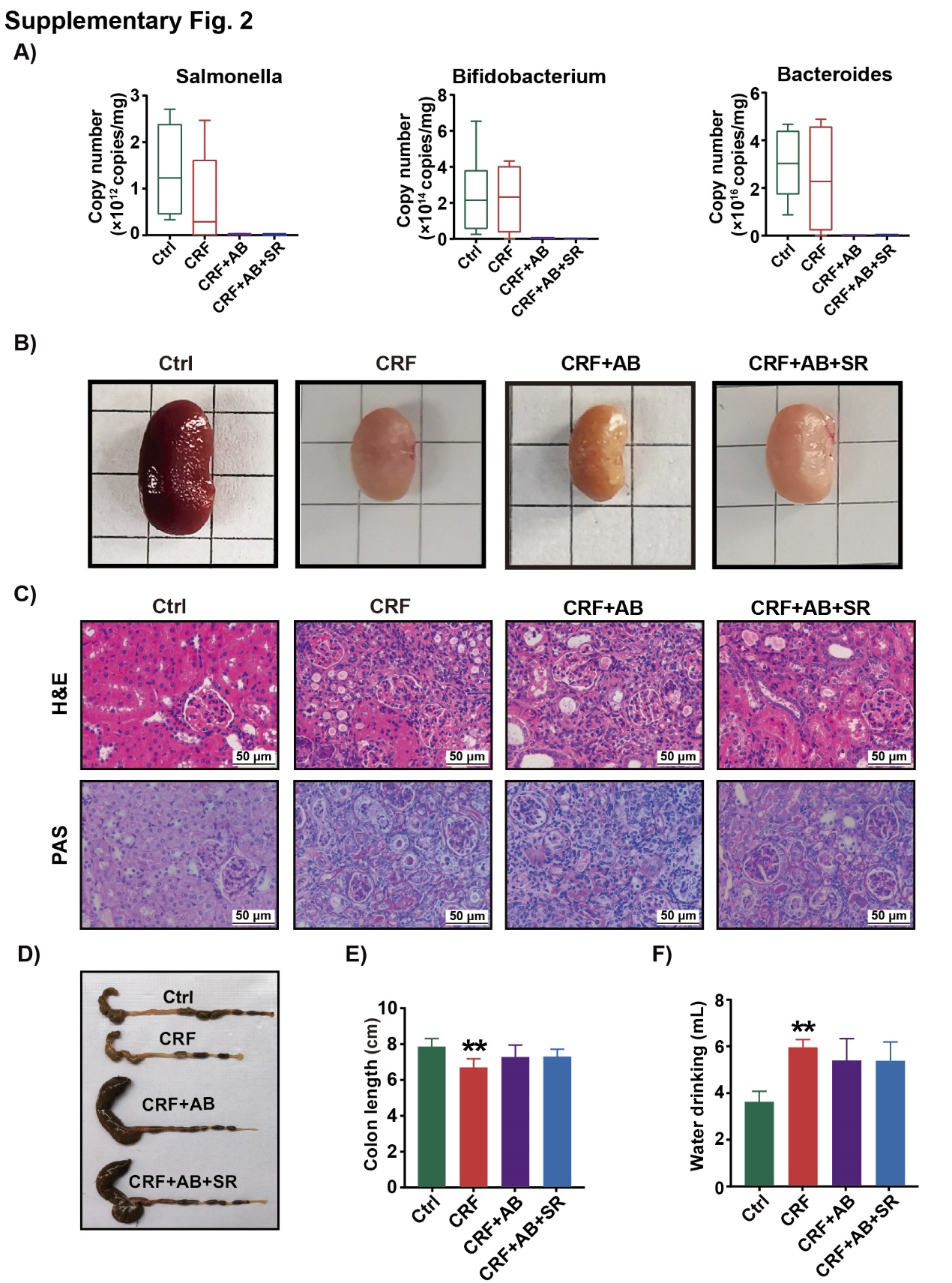


**Supplementary Figure 3.** SR decoction failed to alleviate CRF in mice with gut microbiota depletion. (A) Content assay of Salmonella, Bifidobacterium, and Bacteroides analysis by RT-qPCR. (B) Macroscopic observation of kidney. (C) H&E and PAS staining of kidney tissues (400 ×). (D) Macroscopic observation of colon. (E) Colon length. (F) Water drinking among four experimental groups. Data were presented as mean ± SD. ^**^*P* < 0.01 *vs.* Ctrl group; ^#^*P* < 0.05, *vs.* CRF group.


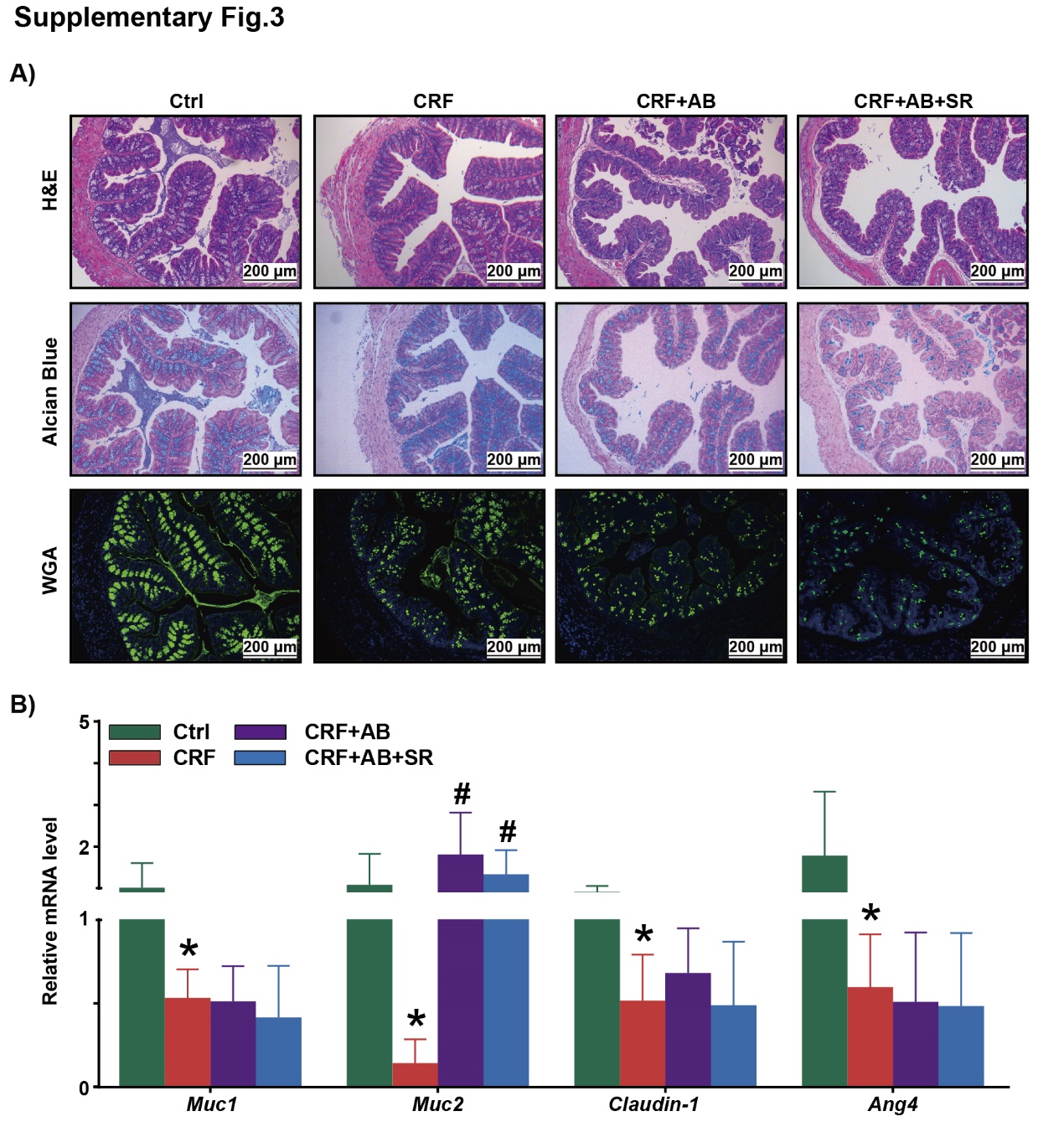


**Supplementary Figure 4.** SR decoction failed to alleviate damage to gut barrier in CRF mice with gut microbiota depletion. (A) Morphology of colon tissues using H&E staining (200 ×), Alcian blue staining (200 ×), and WGA-FITC staining (100 ×). (B) Expressions of gut barrier integrity-related regulators in colon tissues at mRNA levels by RT-qPCR. Data were presented as mean ± SD (n = 6). ^*^*P* < 0.05 *vs.* Ctrl group; ^#^*P* < 0.05 *vs.* CRF group.


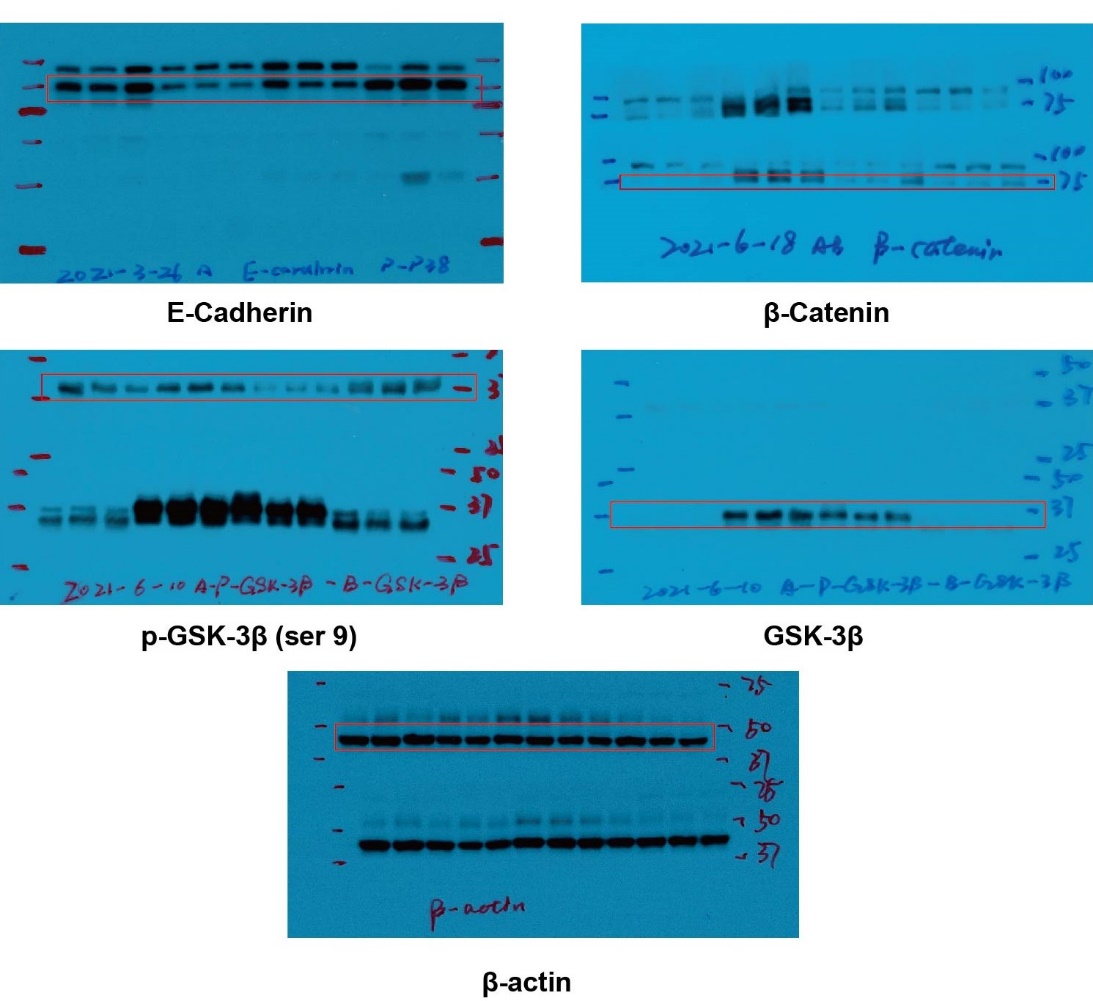


**Supplementary Figure 5.** Uncropped images of original Western blots. Uncropped Western blot results of kidney tissues were provided, including β-Catenin, E-Cadherin, p-GSK-3β (ser 9), GSK-3β, and β-actin.

## Supplementary Tables

**Table 1.** ***Stewed Rhubarb* for the treatment of plasma creatinine and plasma urea in chronic renal failure (mean ± SD)** **(n = 6)**

| Group | Plasma creatinine (mg/dL) | Plasma urea (mg/dL) |
| --- | --- | --- |
| Ctrl group | 1.25 ± 0.19 | 32.18 ± 2.80 |
| CRF group | 3.17 ± 0.34****** | 82.70 ± 5.62****** |
| Dose 1 group（1.0 g/kg·d） | 2.82 ± 0.26 | 70.26 ± 3.90**^##^** |
| Dose 2 group（2.0 g/kg·d） | 2.41 ± 0.33**^##^** | 64.80 ± 3.78**^##^** |
| Dose 3 group（2.5 g/kg·d） | 2.57 ± 0.15**^##^** | 65.95 ± 2.64**^##^** |
| Dose 4 group（3.0 g/kg·d） | 2.66 ± 0.13**^##^** | 67.28 ± 4.93**^##^** |

Note：***P* < 0.01 *vs*. Ctrl group; **^#^***P* < 0.01, *vs*. CRF group.

**Table 2. Primer sequence list for RT-PCR analysis**

| Gene | Forward primer (5'-3') | Reverse primer (5'-3') |
| --- | --- | --- |
| Aqp-1 | GGCGATTGACTACACTGGCT | TGGTTTGAGAAGTTGCGGGT |
| Aqp-2 | TCATCGGTTCCCTCCTCTAC | GTTCCTCCCAGTCAGTGTCC |
| Aqp-3 | ACCCTGCCCGTGACTTTG | ACACCAGCGATGGAACCC |
| Aqp-4 | GCATTTCACTCACGGCTCT | CTCTTGGGAACGGCACTA |
| Claudin-1 | GGGGACAACATCGTGACCG | AGGAGTCGAAGACTTTGCACT |
| Il-1β | GGCTGGACTGTTTCTAATGC | ATGGTTTCTTGTGACCCTGA |
| Muc1 | GGCATTCGGGCTCCTTTCTT | TGGAGTGGTAGTCGATGCTAAG |
| Muc2 | ATGCCCACCTCCTCAAAGAC | GTAGTTTCCGTTGGAACAGTGAA |
| Ang 4 | TTGGCTTGGCATCATAGT | CCAGCTTTGGAATCACTG |
| Nlrp3 | ATTACCCGCCCGAGAAAGG | TCGCAGCAAAGATCCACACAG |
| Tlr4 | GCAGCAGGTGGAATTGTATCG | TGTGCCTCCCCAGAGGATT |
| Tnf-α | GGGTGTTCATCCATTCTC | GGAAAGCCCATTTGAGT |
| Col1a1 | GCTCCTCTTAGGGGCCACT | CCACGTCTCACCATTGGGG |
| Fibronectin | ACGAAGTCAGTGTCTATGC | GAAGCCAGTGATTGTCTCT |
| E-cadherin | GACTTAGAGATTGGCGAATAC | GAGGATGGCAGGAACTTG |
| β-Actin | GGCTGTATTCCCCTCCATCG | CCAGTTGGTAACAATGCCATGT |

**Table 3. Bacterial primer sequences for RT-PCR analysis**

| Bacterium | Forward primer (5'-3') | Reverse primer (5'-3') |
| --- | --- | --- |
| Bacteroides | GGTTCTGAGAGGAGGTCCC | GCTGCCTCCCGTAGGAGT |
| Bifidobacterium | TCGCGTCCGGTGTGAAAG | CCACATCCAGCGTCCAC |
| Salmonella | TGATTGCGATTAGTGC | CTAACGACGACCCTTC |

**Table 4. Quantitative analysis of main compounds in SR decoction by mass spectrometry**

| Compounds | Regression equation | R^2^ | Concentration (mg/mL) |
| --- | --- | --- | --- |
| Gallic acid | y = 1.9138x - 63.7240 | 0.9964 | 11.0500 |
| Rhein-8-O-β-D-glucopyranoside | y = 2.2447x - 56.5550 | 0.9909 | 14.4501 |
| Aloe emodin | y = 0.0050x + 7.2727 | 0.9965 | 11.5501 |
| Rhein | y = 0.0006x + 4.2663 | 0.9970 | 5.0502 |
| Emodin | y = 0.0003x + 4.2668 | 0.9973 | 2.0502 |
| Chrysophanol | y = 0.0021x + 9.1884 | 0.9979 | 3.6002 |
| Physcion | y = 0.0019x + 7.3975 | 0.9990 | 2.2001 |

**Table 5.** **Statistics on raw/clean reads for 16S rDNA sequencing**

| Sample ID | Raw tags | Clean tags |
| --- | --- | --- |
| Ctrl1 | 85085 | 81112 |
| Ctrl2 | 52453 | 51361 |
| Ctrl3 | 60158 | 57687 |
| Ctrl4 | 62482 | 59515 |
| Ctrl5 | 91568 | 88171 |
| Ctrl6 | 76051 | 73184 |
| Ctrl7 | 80447 | 77155 |
| CRF1 | 91264 | 88318 |
| CRF2 | 45003 | 43385 |
| CRF3 | 59257 | 57021 |
| CRF4 | 58633 | 56745 |
| CRF5 | 93066 | 90246 |
| CRF6 | 42081 | 39713 |
| CRF7 | 72755 | 69148 |
| SR1 | 37749 | 36286 |
| SR2 | 68091 | 64330 |
| SR3 | 35982 | 34804 |
| SR4 | 37355 | 36172 |
| SR5 | 60444 | 57980 |
| SR6 | 51315 | 49638 |
| SR7 | 40878 | 39414 |
| CRF+SR1 | 89474 | 85733 |
| CRF+SR2 | 86580 | 82419 |
| CRF+SR3 | 62432 | 59504 |
| CRF+SR4 | 69913 | 67713 |
| CRF+SR5 | 76101 | 71133 |
| CRF+SR6 | 116941 | 111902 |
| CRF+SR7 | 37912 | 35892 |
| Toltal | 1841470 | 1765681 |
